# Tumor-infiltrating immune cell profiles and their change after neoadjuvant chemotherapy predict response and prognosis of breast cancer

**DOI:** 10.1186/s13058-014-0488-5

**Published:** 2014-11-29

**Authors:** Elena García-Martínez, Ginés Luengo Gil, Asunción Chaves Benito, Enrique González-Billalabeitia, María Angeles Vicente Conesa, Teresa García García, Elisa García-Garre, Vicente Vicente, Francisco Ayala de la Peña

**Affiliations:** 1 Department of Hematology and Medical Oncology, University Hospital Morales Meseguer, Murcia, Spain; 2 Department of Pathology, University Hospital Morales Meseguer, Murcia, Spain; 3 Centro Regional de Hemodonación, Murcia, Spain

## Abstract

**Introduction:**

Tumor microenvironment immunity is associated with breast cancer outcome. A high lymphocytic infiltration has been associated with response to neoadjuvant chemotherapy, but the contribution to response and prognosis of immune cell subpopulations profiles in both pre-treated and post-treatment residual tumor is still unclear.

**Methods:**

We analyzed pre- and post-treatment tumor-infiltrating immune cells (CD3, CD4, CD8, CD20, CD68, Foxp3) by immunohistochemistry in a series of 121 breast cancer patients homogeneously treated with neoadjuvant chemotherapy. Immune cell profiles were analyzed and correlated with response and survival.

**Results:**

We identified three tumor-infiltrating immune cell profiles, which were able to predict pathological complete response (pCR) to neoadjuvant chemotherapy (cluster B: 58%, versus clusters A and C: 7%). A higher infiltration by CD4 lymphocytes was the main factor explaining the occurrence of pCR, and this association was validated in six public genomic datasets. A higher chemotherapy effect on lymphocytic infiltration, including an inversion of CD4/CD8 ratio, was associated with pCR and with better prognosis. Analysis of the immune infiltrate in post-chemotherapy residual tumor identified a profile (cluster Y), mainly characterized by high CD3 and CD68 infiltration, with a worse disease free survival.

**Conclusions:**

Breast cancer immune cell subpopulation profiles, determined by immunohistochemistry-based computerized analysis, identify groups of patients characterized by high response (in the pre-treatment setting) and poor prognosis (in the post-treatment setting). Further understanding of the mechanisms underlying the distribution of immune cells and their changes after chemotherapy may contribute to the development of new immune-targeted therapies for breast cancer.

**Electronic supplementary material:**

The online version of this article (doi:10.1186/s13058-014-0488-5) contains supplementary material, which is available to authorized users.

## Introduction

Neoadjuvant chemotherapy (NCT) is an increasingly used therapeutic strategy for early breast cancer. Besides its ability to induce clinical responses that allow breast-preserving surgery [1]-[3], the neoadjuvant setting is a formidable research tool to unveil mechanisms of resistance to treatment. Pathological complete response (pCR) to NCT is currently acknowledged as a surrogate endpoint for therapeutic benefit, especially in human epidermal growth factor receptor 2 (HER2) and basal breast cancer [4]. Schedules that include sequential anthracyclines and taxanes render a higher rate of pCR, thus being the preferred neoadjuvant regimens [5],[6].

Adaptive and innate immune responses play an important role in tumor immunosurveillance, and they may limit the development and growth of neoplasms [7],[8]. The role of immune response in breast cancer is not fully understood, but some recent observations suggest the involvement of tumor microenvironment immune balance in breast cancer response and prognosis. In particular, chemotherapy may trigger an immune response, which contributes to treatment response [9],[10]. Since tumor infiltrating lymphocytes (TILs) are the main actors in the response against cancer cells, they might constitute surrogate markers of the immune balance between the host and the tumor. In breast cancer, the results of studies addressing the issue of tumor immune cell infiltration have consistently demonstrated that a high lymphocytic infiltration predicts a better prognosis [11] and a better response to NCT chemotherapy [12], although this benefit might be restricted to some tumor subtypes. Similarly, the relationship between some subtypes of TIL and breast cancer survival is supported by some studies [13]-[15]. However, conflicting results exist regarding the exact prognostic or predictive value of immune cell infiltrates [16], and the methodological approaches are frequently different among the diverse studies [17]-[19].

In the setting of NCT for breast cancer, mixed results for the diverse TIL subpopulations, such as CD8 or Foxp3, have been found in different studies [13]. Regarding the pre-treatment TIL profile, most studies have evaluated either lymphocytic infiltration as a whole [20]-[22] or a limited set of TIL subpopulations [12],[23]-[25] as predictors of pCR. However, no clinical series in the neoadjuvant setting have included both a broader spectrum of TIL subpopulations and macrophage markers. The changes induced by chemotherapy on TIL populations and the immune profile of the residual tumor, that is, the chemotherapy-resistant tumor, are even less well understood, although they might be more relevant for determining prognosis [12],[13],[24],[25]. Some reports show an increase in TIL (especially CD8) in responding patients [13],[25], and other data point to a decrease of some TIL subpopulations, such as Foxp3 [13], but again no comprehensive evaluations of NCT-induced changes on immune subpopulations are available. Finally, the prognostic impact of the lymphocytic profile change has not been formally evaluated, and the only publication addressing the relevance of post-NCT lymphocytic infiltration in the residual tumor is confined to triple negative breast cancer and does not include data regarding the different lymphocyte sets [26].

The aim of this study was to integrate the predictive and prognostic information obtained from the multiple immune cell populations of breast cancer (CD4, CD8, Foxp3, CD20, CD68), both in the pre-treatment and post-chemotherapy residual tumor setting, and to determine the changes induced by anthracycline and taxane NCT on TIL subpopulations. We here demonstrate that pre- and post-treatment tumor-infiltrating immune cell profiles are able to identify subgroups of patients with different sensitivity to chemotherapy and with different prognosis. CD4 infiltration is identified as the main factor driving these effects. Additionally, chemotherapy-induced changes of immune infiltrates are characterized and their prognostic relevance is shown.

## Methods

### Patients

Clinical data were collected from 121 consecutive patients with stage II or III breast cancer who received NCT in the Department of Hematology and Medical Oncology, University Hospital Morales Meseguer, Murcia, Spain. Clinical evaluation included physical examination, blood tests, chest X-ray, mammography, ultrasound breast exam, breast magnetic resonance imaging (MRI) and core biopsy. Pre-NCT nodal status was determined by axillary and/or supraclavicular ultrasound-guided fine-needle aspiration. In the cases with a negative initial evaluation for nodal metastasis, a sentinel lymph node biopsy (SLNB) was performed before chemotherapy. In locally advanced tumors (defined as cT3N1, cN2-3 or cT4), bone scintigraphy and body computed tomography were added to the staging workup. For chemotherapy response evaluation, dynamic breast MRI was performed prior to surgery. Written informed consent was obtained from all patients and the study was approved by the hospital Institutional Review Board (Comisión de Ensayos e Investigación Clínica, Hospital Morales Meseguer).

### Pathology assessment

Pre-treatment estrogen (ER) and progesterone receptors (PR) status was assessed by immunohistochemistry (IHC), and HER2 status was assessed by either fluorescent *in situ* hybridization (FISH) or a validated IHC method (Herceptest, Dako North America, Inc., Dako, Carpinteria, CA, USA). For ER and PR, cases were considered as negative when the percentage of immunoreactive tumor cells was below 1%; the rest of the cases (≥1% of tumor cells stained) were classified as positive. For HER2, cases were considered positive if Herceptest result was 3+ and/or FISH showed a ratio HER2/CEP17 ≥ 2; the rest of the cases were classified as negative. pCR was defined as the absence of invasive carcinoma both in the breast and the axilla, regardless of the presence of carcinoma *in situ* (ypT0/Tis ypN0). Primary tumor pCR was defined as absence of invasive carcinoma in the breast. Tumors were phenotypically classified according to pre-treatment IHC results as hormone-dependent HER2 negative (ER and/or PR positive and HER2 negative), hormone-dependent HER2 positive (ER and/or PR positive and HER2 positive), HER2 positive (ER and PR negative and HER2 positive) or triple negative (ER negative and PR negative and HER2 negative).

### Treatment

Preoperative chemotherapy included both taxanes and anthracyclines. The NSABP-B27 regimen was the most frequently used and included cyclophosphamide (600 mg/m^2^/21 days) and doxorubicin (60 mg/m^2^/21 days) for four courses, followed by docetaxel (100 mg/m^2^/21 days) for four cycles. After its approval, trastuzumab was administered concomitantly with taxanes to those patients whose tumors overexpressed HER2. Patients treated with docetaxel received prophylaxis with dexamethasone and subcutaneous filgrastim.

After definitive surgery, hormone therapy was administered in all tumors with positive hormone receptors, and, after its approval, adjuvant trastuzumab was given to patients with tumors overexpressing HER2. Adjuvant radiotherapy was administered to all patients treated with breast-preserving surgery and to those patients undergoing mastectomy with any of the following criteria: primary tumor >5 cm, pre or post-chemotherapy T4 and/or N2-3, premenopausal status with pN+, postmenopausal status with pN+ >3, or positive resection margin.

### Tumor-infiltrating lymphocytes assessment

A tissue microarray with paired pre- and post-NCT 2 mm biopsies (two cores for each sample) was built after selection of predominantly tumor areas by a pathologist (ACB). Adequate controls (tonsil and normal breast) were included in each array. For immunohistochemistry, 4 μm sections were cut from the tissue microarray, deparaffinated, rehydrated and processed with standard methods using an automatized stainer (Autostainer Link 48, DAKO, Carpinteria, CA, USA). Secondary antibodies and visualization were performed using standard DAKO Envision systems. Staining was performed simultaneously in all slides to avoid inter-section variability. For TIL study, the following antibodies were used: CD4 (IS649, Dako), CD8 (IS623, Dako), CD3 (IS503, Dako), CD20 (MO755, Dako), FOXP3 (#14-4776, eBioscience, San Diego, CA, USA) and CD68 (IS623, Dako). After assessment of adequate staining by two independent observers, each slide was scanned and digitized with an automated scanning system (Leica SCN400F). Digital images from pre- and post-CT samples were obtained for each tissue core, and after area quantification, tumor area-adjusted morphometric analysis was performed with Image J software (NIH, USA). Results are expressed as TIL count/mm^2^. For each subpopulation, chemotherapy-related relative variation was determined and expressed as a percentage. Using the same tissue microarray, we also performed an evaluation of lymphocytic infiltration based on hematoxylin-eosin staining. Following Denkert’s classification [12], cases were classified in three categories: no lymphocyte infiltrate, partial lymphocyte infiltrate and lymphocyte-predominant breast cancer.

### Hierarchical clustering of lymphocyte markers

In order to conduct an unsupervised hierarchical clustering of the six immune markers, the quantitative values (count/mm^2^) obtained for each case were normalized and categorized in inter-quintile intervals. An average-linkage hierarchical clustering was performed using software Genesis v.1.7.6 [27], primarily designed for analyzing cDNA microarray data, and which also generates a heat map and a dendrogram.

### RNA purification and qRT-PCR assay

Pre- and post-chemotherapy tumor tissues from formalin fixed paraffin-embedded biopsies were deparaffinated with xylene followed by ethanol washes. RNA was extracted with RNeasy FFPE Kit (QIAgen, Germantown, MD, USA) according to the manufacturer’s instructions. Sample retrotranscription and pre-amplification was realized in Mastercycler® nexus (Eppendorf, Hamburg, Germany). Real-time PCR for IFNG and IL10 was performed in LightCycler® 480 System (Roche Diagnostics, Basel, Switzerland) using TaqMan® Gene Expression Assays (Applied Biosystems, Carlsbad, CA, USA). Relative expression levels of each gene were calculated and quantified by the 2^−ΔΔCt^ method using ACTB as endogenous control [28].

### Analysis of public datasets

A group of 1,001 breast cancer patients included in six public genomic datasets (GSE16446 [29], GSE20194 [30], GSE20271 [31], GSE22093 [32], GSE41988 [33] and GSE23988 [34]) were analyzed to confirm the predictive value of pre-chemotherapy immune CD4 and CD8 expression. Patients included were treated with NCT and had available data for pCR and for expression of the six immune markers used in this study (CD3, CD4, CD8, CD20, CD68, Foxp3).

### Statistical analysis

Statistical analysis was carried out with SPSS 20.0 (SPSS, Inc., Chicago, IL, USA). The association between clinical and pathologic parameters was tested with χ^2^ test for categorical variables. Mean differences were studied with the T-test. Disease-free survival (DFS) was measured from the date of diagnosis to the date of last follow-up or disease relapse. Overall survival (OS) was measured from the date of diagnosis to the date of last follow-up or death. Time variables (DFS and OS) were analyzed with the Kaplan-Meier method and groups were compared with the log-rank test. For univariate analysis, the difference between survival functions was calculated using the univariate Cox proportional hazard regression model. Multivariate Cox proportional hazard regression models and logistic regression models were used for multivariate analysis, which included all prognostic factors that were significant in the univariate analysis. For external validation in public datasets, after individual calculation of the odds ratio (OR) for each dataset, we performed a pooled analysis (random-effects model) using the R-based software OpenMetaAnalyst [35].

## Results

### Clinical data and treatment outcomes

One hundred and twenty one breast cancer patients treated with NCT were evaluated. Clinical and pathologic characteristics and treatment data are shown in Table 1. Median age was 56 years (range, 21 to 79 years), and most tumors were stage IIB (28.1%) or IIIA-C (56.4%). Invasive ductal carcinoma was the predominant histology (93.4%) and more than half of the cases were histological grade 3. IHC subtype distribution included 63.6% hormone receptor-positive cases (13.2% HER2 positive and 50.4% HER2 negative), 10.7% HER2 positive hormone receptor-negative and 21.5% triple negative cases. Neoadjuvant treatment mainly consisted of sequential AC-docetaxel (NSABP-B27 schedule) (80.2%), and the pCR rate was 17% (primary tumor pCR: 20.7%; axillary pCR: 26.4%). Tumor subtype (hormone-dependent HER2 negative, hormone-dependent HER2 positive, non-hormone dependent HER2 positive or triple negative) was the only independent predictor of pCR in a logistic-regression multivariate model (OR: 1.87; 95% confidence interval (CI): 1.19 to 2.93; *P* = 0.006). After a median follow up of 60 months neither OS nor DFS has been reached.

**Table 1:** **Patient characteristics**

Characteristic		Number = 121	%
Menopausal status	Premenopausal	60	49.6
	Postmenopausal	61	50.4
Clinical staging	IIA	19	15.7
	IIB	34	28.1
	IIIA	40	33.1
	IIIB	8	6.6
	IIIC	20	16.5
Clinical staging primary tumor	cT1-2	52	43.0
	cT3-4	69	57.0
Clinical staging nodes	cN0	39	32.2
	cN1	36	29.8
	cN2-3	46	38.0
Tumor type	Ductal invasive	113	93.4
	Lobular invasive	5	4.1
	Other	3	2.5
Tumor grade	GI	7	5.8
	GII	39	32.2
	GIII	61	50.4
	Unknown	14	11.5
Hormone-sensitivity	Negative	39	32.2
	Positive	80	66.1
	No data	2	1.7
HER2 overexpression	Negative	87	71.9
	Positive	29	24.0
	Unknown	5	4.1
Triple negative	No	90	74.4
	Yes	26	21.5
	Unknown	5	4.1
IHC subtype	ER+ and/or PR+ and HER2-	61	50.4
	ER+ and/or PR+ and HER2+	16	13.2
	ER- and PR- and HER2+	13	10.7
	ER- and PR- and HER2-	26	21.5
	Non classifiable	5	4.1
Chemotherapy regimen	AC x 4 – Docetaxel x 4	97	80.2
	Sequential anthracyclines-paclitaxel	10	8.3
	Non anthracycline	2	1.7
	Non anthracycline-taxane	4	3.3
	Concomitant anthracyclines–taxane	8	6.6
Trastuzumab treatment	No	99	81.8
	Neoadjuvant	17	14.0
	Adjuvant	5	4.1
Local treatment	Mastectomy	67	55.3
	Breast conservation surgery (BCT)	53	43.8

ER, estrogen receptor, HER2, human epidermal growth factor receptor 2, IHC, immunohistochemistry; PR, progesterone receptor.

### Pre-treatment breast cancer TIL profile and response to chemotherapy

**Figure 1:**
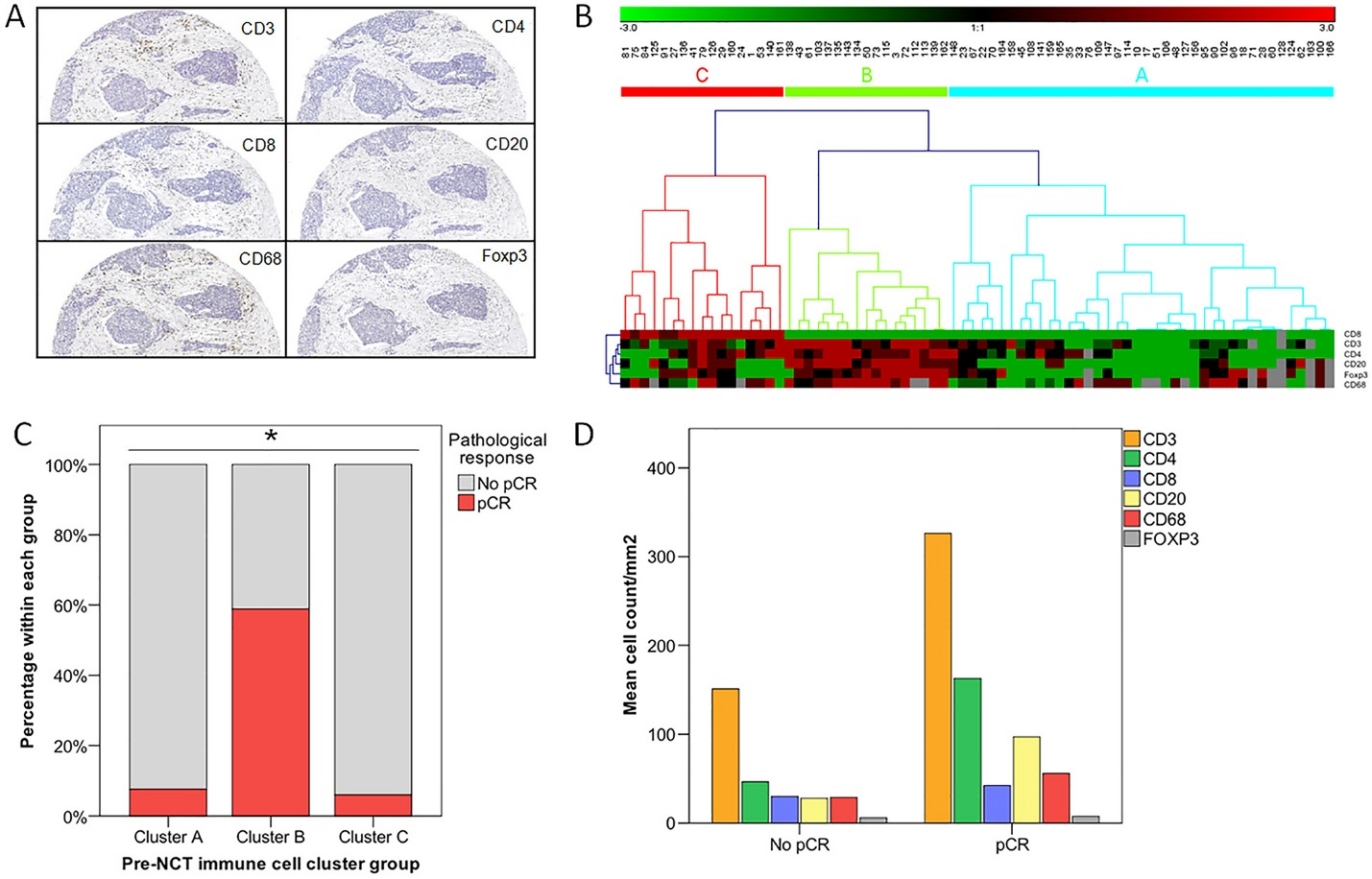
**Pre-treatment lymphocyte subpopulations profile of breast cancer and pathological response to neoadjuvant chemotherapy. A)** representative digital images (100x) of ductal invasive carcinoma of the breast showing CD3, CD4, CD8, CD20, CD68 and Foxp3 staining (scale bar, 100 μm). **B)** unsupervised hierarchical clustering analysis of pre-chemotherapy TIL subpopulations yielded three groups or immune clusters (arbitrarily named A, B and C); each column represents a patient and each row represents an immunohistochemical marker. **C)** pathologic complete response distribution according to pre-chemotherapy immune cluster group. **D)** differential distribution of lymphocyte populations (CD3, CD4, CD8, CD20, Foxp3, CD68) according to pathological response to neoadjuvant chemotherapy; differences were statistically significant for CD3, CD4 and CD20 and non significant for CD8, Foxp3 and CD68. Statistical analysis: Mann–Whitney U-test. *, *P* ≤0.05. TIL, tumor infiltrating lymphocytes.

Tumor material was successfully incorporated in a tissue microarray in 76% of patients (93 of 121) and morphometric quantification by digital imaging analysis was possible in 94% of those cases (Figure 1A). Mean pre-chemotherapy (pre-CT) values of TIL for the whole group are shown in Table 2. CD4 was the predominant lymphocyte subpopulation, with lower values for CD8, while only a small Foxp3 subpopulation was found. Intermediate values were found for B lymphocytes (CD20) and macrophages (CD68) (Additional file 1: Figure S1A). Analysis of percentage distribution showed a predominant CD4 subpopulation over CD8 and CD20 in untreated breast cancer (Additional file 1: Figure S1C). The distribution of T and B cell subpopulations did not differ according to breast tumor characteristics (Additional file 2: Table S1), with the only exception being a higher CD68 infiltration in hormone receptor-negative tumors (*P* = 0.01).

**Table 2:** **Mean pre-chemotherapy counts/mm** ^**2**^ **of tumor infiltrating lymphocytes subpopulations and CD68 for the whole group of patients and comparison between responding and non-responding patients**

TIL/mm ^2^Mean ± SD	Whole group	pCR	Non pCR	***P*** ^***a***^
CD3 cells				
Pre-CT	172.33 ± 334.50	324.33 ± 233.18	138.26 ± 345.70	0.0004
	(n = 71)	(n = 13)	(n = 58)	
Post-CT	100.43 ± 117.43	94.98 ± 158.88	101.74 ± 106.58	0.25
	(n = 88)	(n = 17)	(n = 71)	
Absolute variation	−78.46 ± 360.56	−259.59 ± 249.85	−37.87 ± 370.61	0.001
(post-CT)-(pre-CT)	(n = 71)	(n = 13)	(n = 58)	
Relative variation, %	2459.63 ± 6403.11	281.52 ± 1242.71	2947.83 ± 6978.61	0.002
CD4 cells				
Pre-CT	67.33 ± 123.89	146.94 ± 161.77	48.44 ± 106.28	0.003
	(n = 73)	(n = 14)	(n = 59)	
Post-CT	21.99 ± 69.64	58.38 ± 145.88	13.27 ± 27.66	0.31
	(n = 88)	(n = 17)	(n = 71)	
Absolute variation	−52.28 ± 122.94	−119.93 ± 175.28	−36.23 ± 102.52	0.01
(post-CT)-(pre-CT)	(n = 73)	(n = 14)	(n = 59)	
Relative variation, %	541.63 ± 1817.60	902.63 ± 3590.52	455.97 ± 1082.89	0.03
CD8 cells				
Pre-CT	30.25 ± 101.97	3.58 ± 12.93	36.03 ± 111.65	0.15
	(n = 73)	(n = 13)	(n = 60)	
Post-CT	37.03 ± 66.65	65.61 ± 121.59	30.28 ± 43.95	0.35
	(n = 89)	(n = 17)	(n = 72)	
Absolute variation	3.75 ± 110.74	39.42 ± 46.51	−3.97 ± 119.10	0.08
(post-CT)-(pre-CT)	(n = 73)	(n = 13)	(n = 60)	
Relative variation, %	2650.38 ± 3563.74	4294.12 ± 4085.8	2294.24 ± 3373.26	0.08
FOXP3 cells				
Pre-CT	6.97 ± 17.98	8.37 ± 9.26	6.63 ± 19.53	0.01
	(n = 68)	(n = 13)	(n = 55)	
Post-CT	16.51 ± 85.56	53.73 ± 190.42	7.60 ± 19.86	0.82
	(n = 88)	(n = 17)	(n = 71)	
Absolute variation	0.4859 + 23.03	1.17 ± 27.44	0.32 ± 22.15	0.12
(post-CT)-(pre-CT)	(n = 68)	(n = 13)	(n = 55)	
Relative variation, %	372.91 ± 1317.91	269.25 ± 901.21	397.411 ± 1404.04	0.08
CD20 cells				
Pre-CT	42.08 ± 83.05	92.82 ± 94.10	30.71 ± 76.73	0.000
	(n = 71)	(n = 13)	(n = 58)	2
Post-CT	14.17 ± 53.89	40.83 ± 119.40	7.79 ± 11.70	0.73
	(n = 88)	(n = 17)	(n = 71)	
Absolute variation	−33.86 ± 78.03	−81.31 ± 86.12	−23.23 ± 72.71	0.01
(post-CT)-(pre-CT)	(n = 71)	(n = 13)	(n = 58)	
Relative variation, %	296.45 ± 782.61	−45.09 ± 95.39	373.00 ± 847.17	0.002
CD68 cells				
Pre-CT	33.92 ± 45.09	55.65 ± 65.73	28.60 ± 37.48	0.12
	(n = 61)	(n = 12)	(n = 49)	
Post-CT	39.08 ± 70.77	19.44 ± 23.52	43.51 ± 76.98	0.61
	(n = 87)	(n = 16)	(n = 71)	
Absolute variation	−7.04 ± 58.85	−36.93 ± 62.27	0.27 ± 56.24	0.02
(post-CT)-(pre-CT)	(n = 61)	(n = 12)	(n = 49)	
Relative variation, %	340.13 ± 1132.29	−1.03 ± 159.41	423.68 ± 1249.23	0.16

^a^Mann–Whitney U-test. CT, chemotherapy; n, number; pCR, pathological complete response; SD, standard deviation; TIL, tumor infiltrating lymphocytes.

In order to understand better the complexity of immune subpopulation profiles in breast cancer, an unsupervised hierarchical clustering using the six immune cell markers was performed. Clustering generated three groups of patients arbitrarily designated as cluster A (n = 40; 54%), cluster B (n = 17; 23%) and cluster C (n = 17; 23%) (Figure 1B). A clear association of immune clusters with pCR was found, with cluster B showing a remarkable pCR rate of 58% versus only 7% for clusters A and C (*P* <10^−6^) (Figure 1C). Cluster B group was characterized by low CD8, high CD4, high CD20 and high CD68 infiltration (Table 3), and it showed a significant association with a high histological grade (*P* = 0.03). However, no clear correlation was found between cluster B and age, clinical stage or tumor subtype (Table 4). Multivariate analysis including histological grade and tumor subtype demonstrated the independent predictive value of cluster B for pCR (OR: 18.2; 95%CI: 3.28 to 100.5, *P* = 0.001) after sequential anthracyclines and taxanes NCT. No differences were found for DFS or OS according to pre-treatment immune cluster groups.

**Table 3:** **Distribution of immune cell subpopulations across pre-treatment cluster groups**

	Cluster A	Cluster B	Cluster C
Subpopulation	Mean	Mean	Mean
CD3 pre-CT	51.7	352.0	255.1
CD4 pre-CT	15.7	142.6	110.6
CD8 pre-CT	0.0	0.0	129.9
CD20 pre-CT	4.5	122.0	43.9
FOXP3 pre-CT	3.1	19.3	2.3
CD68 pre-CT	17.5	67.5	34.3

**Table 4:** **Clinical and pathological characteristics of pre-chemotherapy cluster B immune cell profile**

Characteristic		Profile A and C %	Profile B %	***P*** (χ ^2^)
**Age**	<50 years	86	13	0.21
	≥50 years	73	27	
**HER2**	Negative	81	19	0.19
	Positive	66	33	
**Hormone- sensitivity**	No	64	36	0.06
	Yes	83	17	
**IHC subtype**	HR+/HER2-	84	16	0.15
	HR+/HER+	82	18	
	HR-/HER2+	50	50	
	HR-/HER2-	73	27	
**Grade**	G1-2	87	13	0.03
	G3	65	35	
**cN**	cN0-1	81	19	0.24
	cN2-3	69	31	
**cT**	cT1-2	77	23	0.95
	cT3-4	77	23	

HER2, human epidermal growth factor receptor 2; HR, hormone receptor; IHC, immunohistochemistry.

We analyzed the contribution of individual immune cell subpopulations to response to chemotherapy. A significant correlation was found between pCR and higher pre-CT infiltration by CD3, CD4 and CD20 (Table 2): CD3 > 172.3/mm^2^ (*P* = 0.001), CD4 > 67.34/mm^2^ (*P* = 0.001) and CD20 > 42.08/mm^2^ (*P* = 0.001) (Figure 1D). Logistic regression multivariate models including tumor subtype and histological grade confirmed the independent predictive value of a higher pre-NCT CD3, CD4 and CD20 TIL subpopulations for pCR: CD3 (*P* = 0.007; OR = 11.7, 95%CI:1.97 to 69.2); CD4 (*P* = 0.005; OR = 11.0, 95% CI:2.0 to 59.7); and CD20 (*P* = 0.005; OR = 15.3, 95% CI:2.2 to 104.1). Accordingly, a high (over the median) CD4/CD8 ratio was also a stronger and independent predictor of pCR after NCT (*P* = 0.01; OR = 8.5, 95% CI:1.4 to 50.2), thereby partially explaining the predictive value of cluster group B for pCR.

### Analysis of predictive value of CD4 and CD8 expression in public genomic datasets

**Figure 2:**
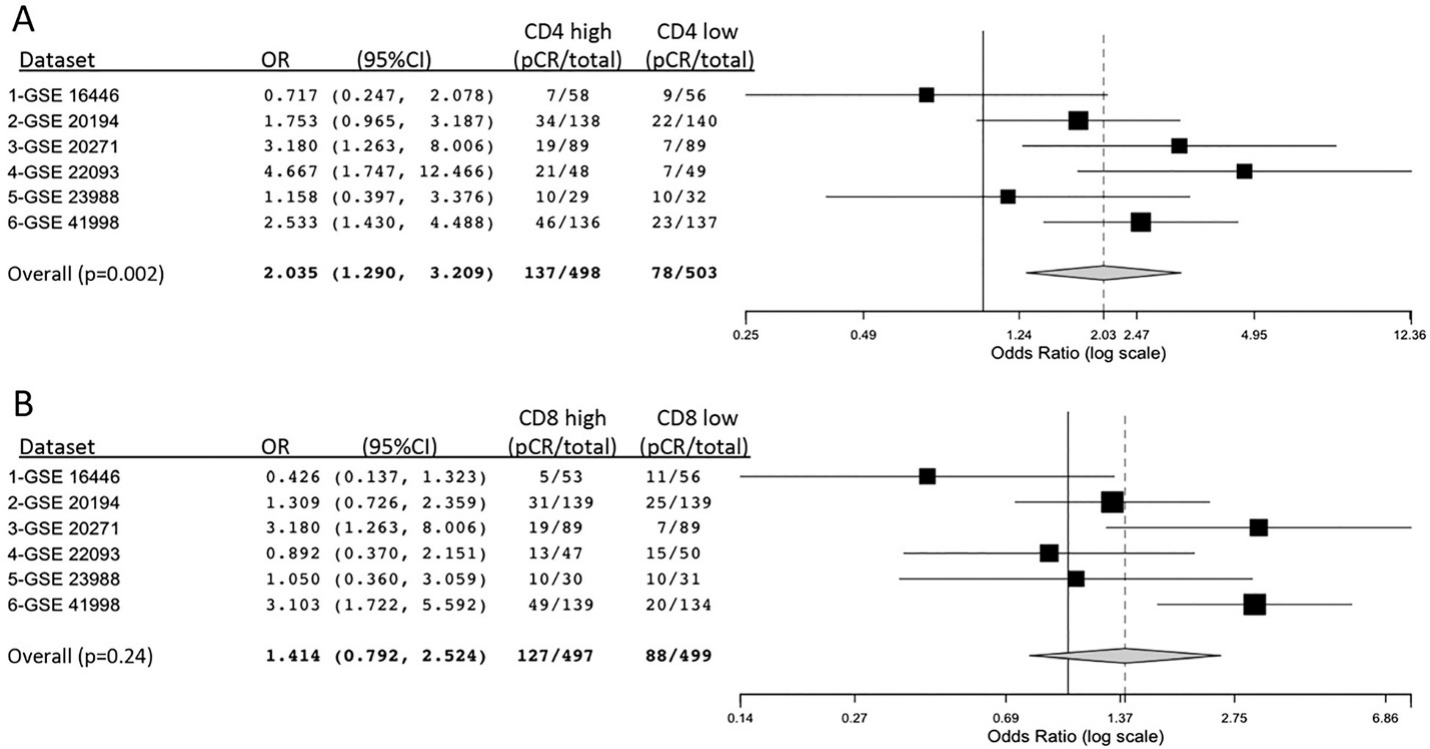
**Analysis of CD4 and CD8 association with response in six genomic public datasets.** Genomic public datasets of breast cancer patients treated with neoadjuvant chemotherapy were analyzed to determine the relative contribution of CD4 and CD8 to pathological response. The odds ratios, with their 95%CI and the proportions of pCR, are shown in the forest plot for each dataset and for the pooled analysis (binary random-effects model); the median value (independently calculated for each dataset) was used as a cut-point between high and low expression. **A)** high CD4 expression was associated (*P* = 0.002) with pCR. **B)** high CD8 expression was more heterogeneous among the six datasets and did not show a significant correlation with pCR after neoadjuvant chemotherapy (*P* = 0.24). pCR, pathological complete response.

In order to externally validate the relative contribution of CD4 and CD8 expression to pCR prediction, we analyzed six public genomic datasets comprising 1,001 patients treated with NCT. Using the median expression as a cut-off for high and low expression, the ORs for each dataset were calculated and a pooled analysis performed according to a random-effects model. As shown in Figure 2, high CD4 expression significantly associated with pCR (OR = 2.03, 95% CI:1.29 to 3.21; *P* = 0.002) while the effect of high CD8 expression on pCR was not clear (OR = 1.41; 95% CI:0.79 to 2.52; *P* = 0.24). In the whole dataset, 27.5% of patients with high CD4 pre-NCT expression obtained a pCR versus only 15.5% of patients with low CD4 expression. CD4 high expression associated with pCR both in high (*P* = 0.05) and low (*P* = 0.0001) CD8 expression groups (Additional file 3: Figure S2A-B), while CD8 expression did not significantly predict pCR in high (*P* = 0.83) and low (*P* = 0.09) CD4 expression groups (Additional file 3: Figure S2C).

### Prognostic value of immune cell infiltration profile in post-chemotherapy residual tumor

**Figure 3:**
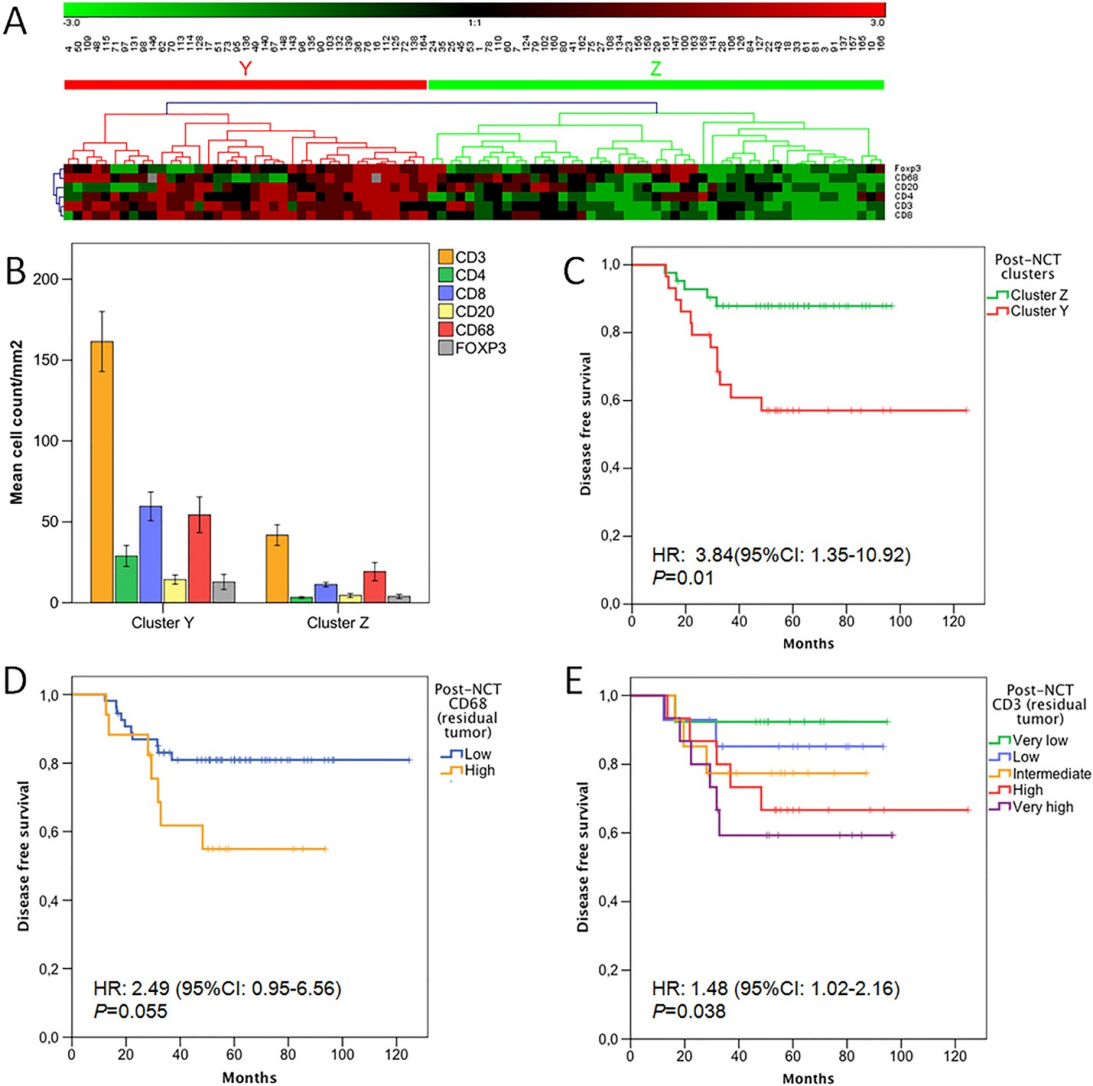
**Post-chemotherapy immune cell subpopulation profile and breast cancer prognosis. A)** unsupervised hierarchical clustering analysis of post-chemotherapy immune cell populations generated two groups (named as Y and Z); each column represents a patient and each row represents an immunohistochemical marker. **B)** distribution of immune cell populations in post-chemotherapy cluster groups Y and Z; statistically significant higher infiltration was shown in cluster Y for all populations (CD3, CD4, CD8, CD20, Foxp3, CD68), Mann–Whitney U-test. **C)** Kaplan-Meier curves showed a worse disease-free survival for patients with tumors belonging to post-chemotherapy immune cluster Y (*P* = 0.01). **D)** disease free survival curves according to post-treatment CD68 infiltration in residual tumor (*P* = 0.055). **E)** Kaplan-Meier disease free survival curves according to level of post-treatment CD3 infiltration in residual tumor, showing an increasingly worse prognosis as CD3 infiltration increases (*P* = 0.038). Hazard ratios (HR) and 95%CI calculated according to Cox proportional hazard regression models.

Mean values of post-NCT immune cell infiltration are shown in Table 2. There was no significant correlation between TIL subpopulations and clinical or pathological characteristics of patients at diagnosis or after NCT (Additional file 2: Table S2). To better appraise the post-treatment tumor immune microenvironment profile, we performed an unsupervised hierarchical clustering analysis of post-treatment immune cell subpopulation distribution in those patients with residual breast carcinoma after chemotherapy. Clustering generated two groups designed as cluster Y and cluster Z (Figure 3A), which basically corresponded with high and low lymphocyte infiltration for all subpopulations (Figure 3B; Table 5). A comparison of clinical and pathological characteristics of both groups showed the association of group Y with histological grade 3 and absence of hormone receptors expression (Table 6). The groups defined by clustering rendered a better prognostic classification than any isolated TIL subpopulation: patients with a residual tumor belonging to cluster group Y showed a worse DFS (*P* = 0.006; log-rank), even after adjusting for post-chemotherapy nodal involvement (*P* = 0.02; HR = 3.38, 95% CI:1.2 to 9.6) (Figure 3C).

**Table 5:** **Comparison of immune cell subpopulations between post-chemotherapy cluster groups (Y versus Z)**

	Cluster Y (post-CT)		Cluster Z (post-CT)		
Subpopulation	Median	SD	Median	SD	***P*** ^a^
CD3 (post-CT)	182.6	117.0	45.9	47.1	<10^−6^
CD4 (post-CT)	27.4	39.3	3.5	3.9	<10^−6^
CD8 (post-CT)	57.9	58.4	11.9	10.2	<10^−6^
CD20 (post-CT)	11.7	13.6	5.1	9.4	0.017
FOXP3 (post-CT)	12.6	28.5	4.2	9.4	0.026
CD68 (post-CT)	62.1	74.6	21.5	42.3	0.001

^a^Mann–Whitney U-test. CT, chemotherapy; SD, standard deviation.

**Table 6:** **Comparison of clinical and pathological characteristics of post-CT immune cell clusters (Y and Z) in BC patients with residual tumor after NCT**

		Number = 71				
		Cluster Y		Cluster Z		***P*** (χ ^2^)
Characteristic		Number	%	Number	%	
Menopausal status	Postmenopausal	10	34.5	23	54.8	0.09
	Premenopausal	19	65.5	19	45.2	
Tumor type	Ductal invasive	27	93.1	40	95.2	0.70
	Lobular invasive	2	6.9	2	4.8	
	Others	0	0.0	0	0.0	
Grade 3 (pre-CT)	Grade 1-2	7	28.0	26	65.0	0.004
	Grade 3	18	72.0	14	35.0	
Grade 3 (residual tumor)	Grade 1-2	15	51.7	29	74.4	0.053
	Grade 3	14	48.3	10	25.6	
HER2	HER2 negative	22	78.6	35	83.3	0.61
	HER2 positive	6	21.4	7	16.7	
Hormone-sensitivity	Negative	12	42.9	7	16.7	0.016
	Positive	16	57.1	35	83.3	
IHC subtype	HS	12	42.9	31	73.8	0.033
	HS-HER2+	4	14.3	4	9.5	
	HER2+	2	7.1	3	7.1	
	Triple negative	10	35.7	4	9.5	
cN2-3 (pre-CT)	cN0-1	19	65.5	32	76.2	0.32
	cN2-3	10	34.5	10	23.8	
ypN	ypN0	12	41.4	21	50.0	0.47
	ypN+	17	58.6	21	50.0	
ypN2-3	ypN0-1	17	70.8	26	76.5	0.63
	ypN2-3	7	29.2	8	23.5	
cT3-4 (pre-CT)	cT1-2	12	41.4	20	47.6	0.60
	cT3-4	17	58.6	22	52.4	
ypT	ypT0/is	0	0.0	0	0.0	0.43
	ypT1	10	34.5	9	21.4	
	ypT2	12	41.4	19	45.2	
	ypT3	4	13.8	11	26.2	
	ypT4	3	10.3	2	4.8	
	ypTx	0	0.0	1	2.4	

BC, breast cancer; CT, chemotherapy; HER2, human epidermal growth factor receptor 2; IHC, immunohistochemistry.

To identify the contribution of each immune cell subpopulation to the biological behavior of post-treatment breast tumors, we analyzed their prognostic impact both in the whole group and in those patients with residual (resistant to chemotherapy) tumor (Additional file 2: Table S3). Differences were mainly found for CD68, a marker of tumor associated macrophages, which have been linked to tumor progression and worse prognosis both in experimental models and clinical series [36]. For the whole group of patients (with or without pCR) we observed a worse DFS and OS in those cases with higher CD68 infiltration after chemotherapy (*P* = 0.03), which was lost in multivariate analysis. The prognostic effect of post-chemotherapy CD68 infiltration was especially observed in those patients with residual tumor (no pCR), in whom a higher CD68 count trended to associate with worse DFS in univariate (*P* = 0.055) and multivariate analysis (*P* = 0.09; HR = 2.26, 95% CI: 0.86 to 5.96) (Figure 3D). A gradual prognostic effect of tumor-infiltrating CD3 was also observed in those cases with residual carcinoma, with higher infiltration associated with worse DFS in the univariate analysis (*P* = 0.038) (Figure 3E).

Since previous reports point to a better prognosis for those patients showing very high TIL in residual tumor [26], we next tested whether post-NCT immune cluster classification might just rely on the level of TIL in post-treatment tumor. Survival analysis stratified by high (P75) CD3 infiltration, a likely surrogate of high TIL, showed that immune cluster classification kept its statistical significance for DFS (*P* = 0.008, log-rank), while only a trend was observed for OS (*P* = 0.16, log-rank) (Additional file 4: Figure S3).

Finally, we explored the possibility of a differential effect of post-treatment immune infiltrate in residual tumor across the different breast cancer subtypes (Additional file 2: Table S4). A higher deleterious effect of CD68 infiltration was found in HER2 tumors (*P* = 0.04), while in hormone receptor-positive tumors the main factor associated with worse DFS was a higher CD8 count (*P* = 0.04).

### Comparison of hematoxylin-eosin-based lymphocytic infiltration classification with immunohistochemistry-based immune cell profiles

In order to determine if pre-NCT and post-NCT cluster group classification added any information to the HE-based morphological evaluation of tumor-infiltrating lymphocytes, we categorized breast tumors according to Denkert’s classification (no infiltrate, partial infiltrate, lymphocyte-predominant breast cancer). As shown in Additional file 2: Table S5, both the mean number of CD3 cells and the total number of immune cells (CD3 + CD20 + CD68) showed a good correlation with the HE-based lymphocyte infiltrate classification.

Pre-chemotherapy TILs, as determined by conventional histology, showed a good predictive value for pCR (OR:4.8; 95% CI: 1.6 to 14,6; *P* = 0.006). However, this predictive value was lost in a multivariate model in which both the clinical covariates (histological grade and tumor subtype) and the cluster B were introduced. In the final model for pCR, only tumor subtype (OR: 3.8; 95% CI: 1.2 to 12.1; *P* = 0.02) and cluster B (OR: 28.5; 95% CI:2.1 to 390.4; *P* = 0,01) were statistically significant.

The prognostic value of HE-determined TIL was also evaluated both in the pre- and post-treatment setting. Differences were found neither for DFS (log-rank; *P* = 0.27) nor for OS (log-rank; *P* = 0.30) according to pre-chemotherapyTIL morphologic classification. In the group of patients with post-chemotherapy residual tumor, no statistically significant differences were observed for DFS (log-rank; *P* = 0.33) or OS (*P* = 0.18). After inclusion of post-NCT HE-determined lymphocytic infiltration together with post-treatment cluster (Y versus Z) in a Cox model for DFS, only the post-treatment cluster group kept the prognostic value (HR: 3.3; 95% CI: 1.1 to 9.8; *P* = 0.03).

### Changes induced by chemotherapy on breast cancer immune cell infiltration

We evaluated the pattern of treatment-induced changes in immune cells infiltration. NCT produced a statistically significant decrease of CD4 (*P* = 0.01), CD20 (*P* = 0.04) and CD68 (*P* = 0.03) cell counts (Table 2). Changes in CD8 infiltrate occurred in the opposite direction, with a clear increase after chemotherapy (*P* = 0.0001), while Foxp3 remained unchanged (*P* = 0.86). Although post-treatment total CD3 counts were lower, this difference did not reach statistical significance (*P* = 0.47). Taken together these data support a chemotherapy-induced change in the distribution of lymphocyte subpopulations, with an inversion of the CD4/CD8 ratio and a decrease of B cells and macrophage infiltration after treatment.

**Figure 4:**
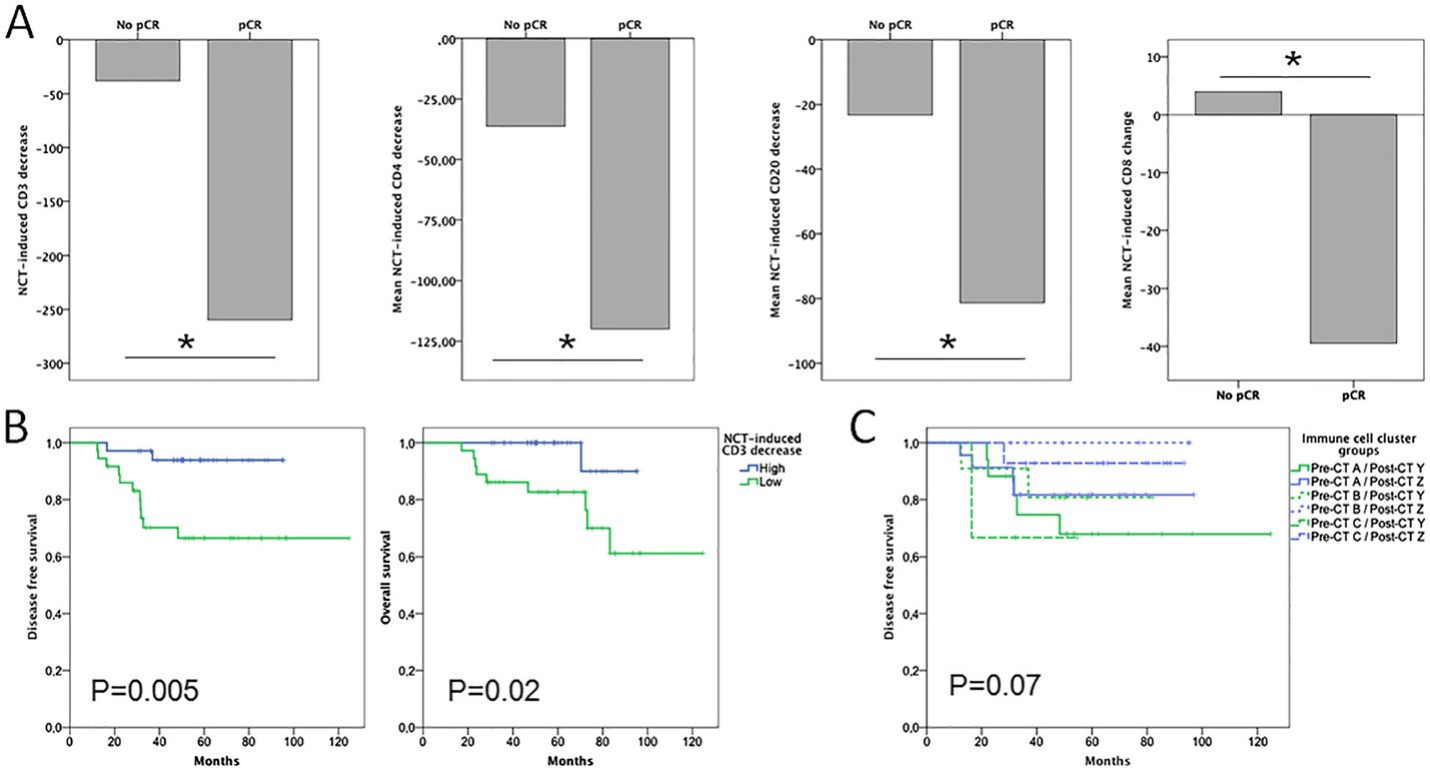
**Association of pCR and prognosis with chemotherapy-induced changes on breast cancer immune cell subpopulations. A)** significant association of higher chemotherapy-related changes of CD3, CD4, CD20 and CD8 populations with pCR; chemotherapy-induced decrease of CD3, CD4 and CD20 was significantly higher in those patients achieving pCR. Statistical analysis: Mann–Whitney U-test. *, *P* ≤0.05. **B)** Kaplan-Meier disease free and overall survival curves according to chemotherapy-induced CD3 decrease (log-rank test). **C)** Kaplan-Meier showing the prognostic effect of post-chemotherapy cluster group (Y-Z) among the three pre-treatment cluster groups **(A-C)**, and supporting that the prognostic impact of post-treatment immune cell profile was independent of the baseline immune cell cluster group (stratified log-rank test). pCR, pathological complete response.

The degree of TIL distribution changes exerted by chemotherapy was related to pathological response (Figure 4A): a higher (over the median) chemotherapy-induced decrease of the total infiltration by T cells (CD3) was significantly related to pCR even after adjusting for tumor subtype in the multivariate analysis (*P* = 0.001; OR = 17.84, 95% CI:3.02 to 105.27). This tumor subtype-independent association between pCR and a higher effect of chemotherapy on tumor infiltration was also found for two particular lymphocyte subpopulations: CD4 (*P* = 0.001; OR = 15.02, 95% CI: 2.89 to 77.92) and CD20 (*P* = 0.002; OR = 11.87, 95% CI: 2.47 to 57.01) (Additional file 2: Table S6). The modulation of the TIL profile by chemotherapy not only was associated with response, but also with survival: higher CD3 decrease after treatment was related to better OS (*P* = 0.02) and DFS (*P* = 0.005) (Figure 4B), although only a trend for DFS was kept in the multivariate analysis (*P* = 0.08; HR = 4.5, 95% CI: 0.8 to 24.9).

Finally, we evaluated the evolution from pre-treatment immune cluster A-C groups to post-chemotherapy Y-Z cluster groups in those patients with residual tumor. The transition between pre-NCT and post-NCT immune cluster groups was unequally distributed, with more patients from group A (40.5%) and B (71.4%) evolving to group Y (*P* = 0.05) when compared with cases included in group C (18.8%). However, the prognostic relevance of post-NCT immune cluster groups was apparently independent of the pre-NCT group (A, B or C) as suggested by the stratified DFS analysis (*P* = 0.07; log-rank) in which the deleterious prognostic impact of cluster Y was observed among all pre-treatment clusters groups (Figure 4C).

### Expression of immune response mediators according to immune cell infiltration profiles

In order to determine the functional basis of immune cell infiltration profiles we analyzed the mRNA expression levels of IFNG, a marker of Th1 response, and IL10, a Th2 mediator, by RT-qPCR in pre- and post-treatment breast biopsies. IFNG expression, classified as high or low (over or under the median value), was significantly different between clusters (χ^2^, *P* = 0.025), with more cases in clusters B and C showing high expression, while most tumors (65%) included in cluster A had low IFNG expression (Additional file 5: Figure S4A). IL10 expression showed a different pattern, with virtually no cases of pre-NCT profile B showing a high level (P75) of IL10 expression (1/15 cluster B versus 13/43 cluster A-C; Fisher’s exact test, *P* = 0.087) (Additional file 5: Figure S4B-C). Mean expression levels were not significantly different in any of the pre-treatment cluster groups (Additional file 5: Figure S4D-E). High expression of IFNG was also associated with pCR (30.4% versus 11.1% pCR; *P* = 0.023), while the association was unclear for low IL10 expression (*P* = 0.22) (Additional file 5: Figure S4F-G). Taken together, these data suggest that cluster group B might be characterized by a lower level of IL10 expression in the context of a high IFNG expression, which is consistent with a dominant Th1 response profile.

## Discussion

Immune response in breast cancer has been recently recognized as a potential mechanism mediating tumor progression and response to treatment. Analysis of baseline tumor infiltrating lymphocytes (TIL), a likely surrogate of the immune balance in the tumor microenvironment, has shown prognostic value in large clinical series of breast cancer [11],[17],[37]. However, the contribution of the different TIL subpopulations to the clinical and biological behavior of the tumor is still unclear. Lymphocytic infiltration in breast tumor has also been shown to be a potent predictive factor for response in patients undergoing NCT for breast cancer [12],[20], although further knowledge of the detailed effects of chemotherapy on breast cancer immune response is needed. In this work, we show that the pre-treatment profile of immune cell subpopulations is able to identify a highly responsive group of breast carcinomas characterized by a high CD4, CD68 and CD20 and a low CD8 infiltration. We also analyze the effects of chemotherapy on TIL subpopulations and the immune profile of post-NCT residual tumor. Our results support the prognostic impact of chemotherapy-mediated immune changes and identify a high-risk post-NCT tumor immune cell profile.

Although lymphocytic infiltration is emerging as a potential prognostic factor in breast cancer, its evaluation has not been standardized yet. In a large adjuvant series of breast cancer, lymphocytic predominance was defined as infiltration over 50% [11]. Other studies have used either quantitative or semiquantitative methods [12] for TIL evaluation with variable cut-points used to define low and high lymphocytic infiltration. All these methodological approaches depend on manual counting by a pathologist, and are time-consuming and difficult to standardize. Few groups have used computer-based evaluation of breast cancer TIL infiltration, which is especially feasible when combined with IHC detection of lymphocytes [12],[38].

Our choice of an open-source software analysis of digital images for quantifying breast cancer TIL and CD68 was also undertaken to avoid inter-observer bias and to favor the future standardization of TIL evaluation. Evaluation of the whole sample, including both the tumor and the peritumor stroma, further simplifies image processing and is supported by previous results showing that intratumoral and peritumoral lymphocytic infiltration have a similar prognostic value [11],[39]. The results shown here for CD3, a pan-T cell marker, are consistent with previous extensive data on the predictive value for pCR of high TIL in breast cancer [12],[20] and the correlation of CD3 with manually-counted TIL [12], thereby supporting the feasibility of an IHC-based automated method to evaluate lymphocytic infiltration in breast cancer. The comparison of these results with the HE-based morphological classification of lymphocyte infiltration also supports the value of computer-based methods. However, extensive work is still needed to validate and standardize these methods before introducing them in the clinical setting.

Previous studies of TIL in breast cancers have addressed the prognostic or predictive relevance of individual immune cell subpopulations, showing mixed or conflicting results. High CD3, CD20 [12] and CD8 in the tumor have been variably identified or excluded as predictive factors for pCR [13],[25]. Other authors have used definition of TIL ratios, such as CD8/CD4 [38] or Foxp3/CD3 [40], as an alternative approach to better integrate the information provided by each TIL subpopulation. Our evaluation was, instead, based on the unsupervised hierarchical clustering of all subpopulations across the whole group of patients. The underlying assumptions of this strategy are the complexity of immune balance in the tumor microenvironment and the potential contribution of each immune cell subpopulation to determine the immune response against the tumor and the level of cooperation with chemotherapy effects. Our results support this type of approach and allowed us to identify a subgroup of patients highly sensitive to anthracyclines and taxanes NCT, independently of grade or tumor subtype.

Cluster B pre-NCT group showed an extremely high response to chemotherapy, with 58% of patients achieving a pCR. This level of pCR for the responsive group is higher than those reported by other groups using hematoxylin-eosin methods [20], IHC-based manual counting [12],[21] or even immune-related signatures [41],[42]. The characteristics of this group (CD4 high, CD8 low, CD20 high and CD68 high) are not totally coincident with previous results in the literature and require further explanation. For a long time, cytotoxic CD8 lymphocytes have been considered the main mediators of tumor immune surveillance, while CD4 lymphocytes either have not been evaluated or have been associated with suppression of anti-tumor immune response. De Nardo *et al*. showed experimental and clinical data consistent with this paradigm in breast cancer [38], although their analysis of the CD68/CD8 profile as a predictor of pCR was based on fine-needle aspiration (FNA) samples, in which stroma and immune-related stromal components are usually underrepresented in comparison with the core biopsy samples obtained from patients in our series [43]. Other work has shown the predominance of the CD4 population over CD8 in breast cancer, and has suggested that CD8 cytotoxicity is minimal in untreated tumors [44]. Recent data also support a more complex functional role of CD4 cells in breast cancer than considered before: extensive lymphocytic infiltration of breast cancer has been linked to increased CD4+ Th1 and Tfh populations, and is associated with better survival and higher pCR rates [45]. Other work also suggests that low CD4 counts in the tumor [46] or in peripheral blood [47] are a negative prognostic factor in early or metastatic breast cancer. Finally, results might also differ between adjuvant and neoadjuvant series, perhaps pointing to a potential interaction of the tumor immune profile and its modulation by exposure to chemotherapy.

Changes induced by chemotherapy on breast cancer lymphocytic subpopulations have received less attention and contradictory data have been reported. Similarly to our results, De Maria *et al*. found that increased TIL, specifically CD8, associated with pCR. Ladoire *et al*., using a semiquantitative scoring of TILs, showed the association of pCR with the disappearance of Foxp3 TIL; similarly to our results, they did not find significant NCT-induced changes on CD3 counts. However, they did not observe changes in CD8 infiltrate, which was increased after NCT in our series; no evaluation of CD4, CD20 or CD68 was performed [13]. Reasons for these discrepancies are unclear, but a contribution of the chemotherapy schedule cannot be ruled out since immune effects differ between different drugs [48] and most previous reports used anthracyclines as the main treatment regimen and the percentage of sequential anthracyclines and taxanes was low (20% in the series from Ladoire versus 88% in ours). Interestingly, a comprehensive analysis of the immune characteristics of a small group of breast carcinomas also showed an increase of CD8 and a decrease of CD4 and CD20 lymphocytes after chemotherapy [44].

The analysis of the immune cell pattern in post-chemotherapy residual breast cancer might provide a better prognostic stratification of this poor prognosis group, and contribute to identify subgroups of patients amenable to therapeutic strategies targeting the tumor immune response. The unsupervised clustering approach performed in patients without primary tumor complete response was able to identify two clearly differentiated prognostic groups (post-NCT clusters Y and Z). Paradoxically, the post-NCT group with a higher TIL presence (cluster group Y) showed a significantly worse DFS. These findings might be at least partially explained by the presence of a predominant infiltration by CD68, a macrophage marker previously associated with tumor progression and distant recurrence [38],[49], and also with a trend to worse DFS in our series. The identification of these high-risk patients, especially in the group with voluminous residual disease, might lead to adjuvant immune treatments such as agents modulating tumor associated macrophages. Our data might be seen as opposite that of a recent report by Dieci *et al*. showing that high TIL (as evaluated by HE) in post-NCT residual triple negative breast tumor is a predictor of good prognosis [26]. However, given the sample size and the high pCR of triple negative breast cancer (TNBC) primary tumor in our series, we only identified three patients with TNBC and residual tumor with high TIL infiltration (defined as CD3 counts over percentile 75). Our results showing that post-NCT immune cluster groups are able to prognostically classify patients even after stratifying by high CD3 counts suggest that TIL subpopulation analysis might further refine the selection of high risk patients among those with high TIL in non-TNBC residual tumor, but the characteristics of the sample do not allow us to sustain the same conclusion for TNBC with residual disease.

Our work has some limitations, the main one being the limited sample size. This fact precluded a more extensive analysis of the interactions between the immune response profile and the tumor subtype. However, other work has shown that prediction of pCR by immune-related signatures is probably not confined to HER2 or triple negative tumors and may also be reliable in luminal breast cancer [42]. Our analysis of the immune profile of residual tumor mainly includes hormone receptor-positive tumors (77%), a group in which the prognostic impact of pathologic response is limited [4] and in which a better prognostic stratification is particularly needed. A second limitation is the lack of standardization and external validation of our computer-based method for immune cell evaluation and the potential sampling bias induced by the use of tissue microarrays instead of full sections, especially in the post-chemotherapy setting. Finally, a third limitation common to other TIL studies, is that the evaluation of tumor-infiltrating immune cells is probably only an imperfect surrogate of the type of immune response in the tumor microenvironment. Besides methodological variability, morphological and immunohistochemical data are difficult to understand functionally. Our results regarding IFNG and IL10 expression should be considered merely exploratory, and other experimental approaches, such as detailed cytometry of functional subpopulations [44] or immune mediators expression arrays [42], are warranted to obtain further insights on the clinical relevance of breast cancer immune balance. However, our finding of a predominantly low IL10 expression combined with high IFNG expression in the highly responsive cluster group B, might be consistent with a high Th1/Th2 balance, a known marker of an appropriate anti-tumor immune response [50]. Although limited by the categorical analysis and the lack of evaluation of other Th1/Th2 mediators, these data, taken together with the results showing that those patients with a larger immune modulation by NCT also had a better response, might support the cooperation of immune response with chemotherapy anti-tumor effects [10].

## Conclusions

An IHC-based profile of immune cell subpopulations in breast cancer is able to identify a group of tumors highly sensitive to NCT. This morphometric approach seems technically feasible and might be preferable to traditional TIL evaluation. The study of chemotherapy-related immune changes and the lymphocytic subpopulation profile of post-NCT residual tumor also allows prognostic stratification of this group of high-risk patients independently of nodal residual disease. Further research of the mechanisms underlying these findings may ease the pathway for developing new immunity-targeted therapeutic strategies in breast cancer patients.

## Additional files

## Electronic supplementary material

Additional file 1: Figure S1.: Distribution of individual immune cell populations in pre- and post-treatment breast cancer biopsies. A) pre-chemotherapy distribution of immune cell populations (absolute counts/mm^2^). B) post-chemotherapy distribution of immune cell populations (absolute counts/mm^2^). C) percentage distribution of immune cell populations in pre- and post-treatment biopsies, showing a chemotherapy-induced inversion of CD4/CD8 ratio and a decrease of CD20; percentages calculated over total immune cell counts (CD4 + CD8 + CD20 + Foxp3 + CD68). (TIFF 663 KB)

Additional file 2: Table S1: Distribution of TIL subpopulations according to breast tumor characteristics. **Table S2.** Association of post-chemotherapy immune cell infiltration with clinical and pathological characteristics. **Table S3.** Association of post-chemotherapy immune cell subpopulations with breast cancer disease-free (DFS) and overall survival (OS). **Table S4.** Prognostic effect (DFS) of post-treatment immune infiltrate in residual tumor according to tumor subtypes. **Table S5.** Association of HE-based classification of lymphocyte infiltration and immunohistochemistry-based assessment of CD3 and other immune cell subpopulations. **Table S6.** Impact of chemotherapy-induced changes (high versus low) on pCR (multivariate model including tumor phenotype). (PDF 176 KB)

Additional file 3: Figure S2: Relative contribution of CD4 and CD8 expression to pCR in public genomic datasets. A) distribution of cases among the whole series (n = 1001 patients; genomic datasets: GSE 16446, GSE 20194, GSE 20271, GSE 22093, GSE 23988, GSE 41998) according to high or low expression of CD4 and CD8 (cut-point: median value); pCR rates are shown for each subgroup. B) CD4 expression was associated with pCR both in the CD8 low group (*P* = 0.0001) and in the high CD8 group (*P* = 0.05). C) significant association of pCR with CD8 was found neither in the low CD4 group (*P* = 0.09) nor in the high CD4 expression group. Statistical analysis: χ^2^ test. *, *P* ≤0.05. NS, non significant. (TIFF 1 MB)

Additional file 4: Figure S3: Disease free and overall survival analysis of post-NCT clusters (Y and Z) stratified by lymphocytic (CD3) infiltration. Kaplan-Meier curves showing the prognostic impact of post-NCT tumor-infiltrating immune cell profiles in tumors with low and high lymphocytic infiltration (defined as CD3 under or over P75). The difference was significant for DFS (*P* = 0.008; log-rank stratified by CD3 infiltration), while only a trend was found for OS (*P* = 0.16; log-rank). Stratified Wilcoxon test was significant both for DFS (*P* = 0.03) and OS (*P* = 0.03). (TIFF 1004 KB)

Additional file 5: Figure S4: Pre-chemotherapy IFNG and IL10 expression. A) pattern of IFNG level of expression (over or below the median value) according to pre-NCT clusters (*P* = 0.025), B-C) pattern of IL10 expression (P75) according to pre-NCT clusters (*P* = 0.116; A-C versus B, *P* = 0.087). D) box-plot showing the level of IL10 expression among pre-treatment cluster groups (A-C); expression levels normalized to lower group (NS). E) level of IFNG expression according to pre-NCT cluster group (NS). F) association of IFNG level of expression (over or below median) with pCR (*P* = 0.023). G) association of IL10 expression (over or below P75) with pCR (NS). Statistical analysis: Mann–Whitney U-test or χ^2^ test . NS, non significant. *, *P* ≤0.05. (TIFF 1 MB)

Below are the links to the authors’ original submitted files for images.

Authors’ original file for figure 1

Authors’ original file for figure 2

Authors’ original file for figure 3

Authors’ original file for figure 4

## Abbreviations

BC: breast cancer
CI: confidence interval
CT: chemotherapy
DFS: disease free survival
ER: estrogen receptor
HE: hematoxylin-eosin
HER2: human epidermal growth factor receptor 2
HR: hormone receptors
IFNG: interferon G
IHC: immunohistochemistry
IL10: interleukin 10
MRI: magnetic resonance imaging
NCT: neoadjuvant chemotherapy
OR: odds ratio
OS: overall survival
pCR: pathological complete response
PR: progesterone receptor
SLNB: sentinel lymph node biopsy
TIL: tumor-infiltrating lymphocytes
TNBC: triple negative breast cancer
