# Supplementary figures and images for: Tumor-infiltrating immune cell profiles and their change after neoadjuvant chemotherapy predict response and prognosis of breast cancer
Source: Breast Cancer Res. 2014 Nov 29;16:488. doi: 10.1186/s13058-014-0488-5 (PMC4303200; doi:10.1186/s13058-014-0488-5)

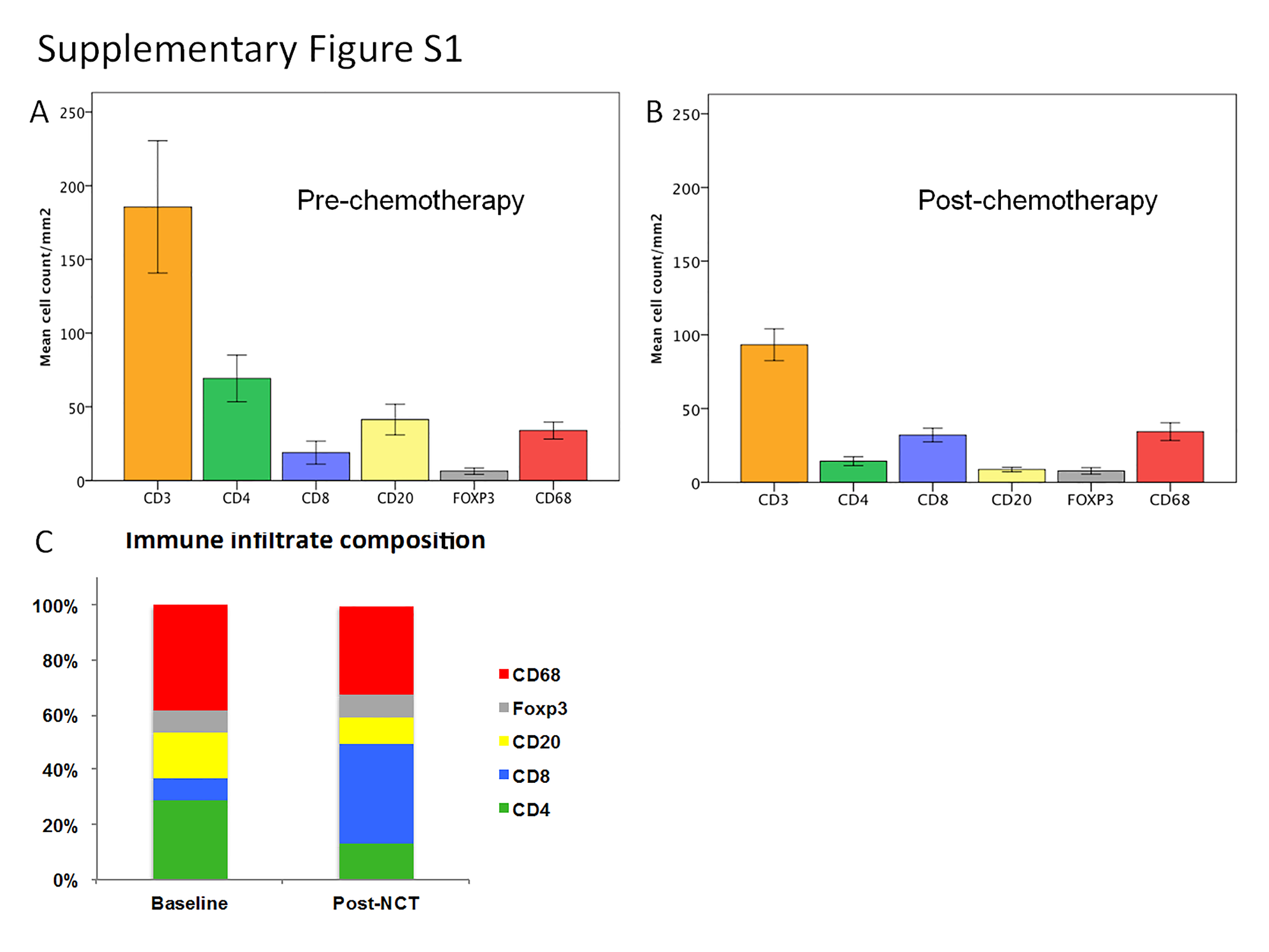

Supplement: Supplementary file 1 — Additional file 1: Figure S1.: Distribution of individual immune cell populations in pre- and post-treatment breast cancer biopsies. A) pre-chemotherapy distribution of immune cell populations (absolute counts/mm2). B) post-chemotherapy distribution of immune cell populations (absolute counts/mm2). C) percentage distribution of immune cell populations in pre- and post-treatment biopsies, showing a chemotherapy-induced inversion of CD4/CD8 ratio and a decrease of CD20; percentages calculated over total immune cell counts (CD4 + CD8 + CD20 + Foxp3 + CD68). (TIFF 663 KB) [file 13058_2014_488_MOESM1_ESM.tiff]

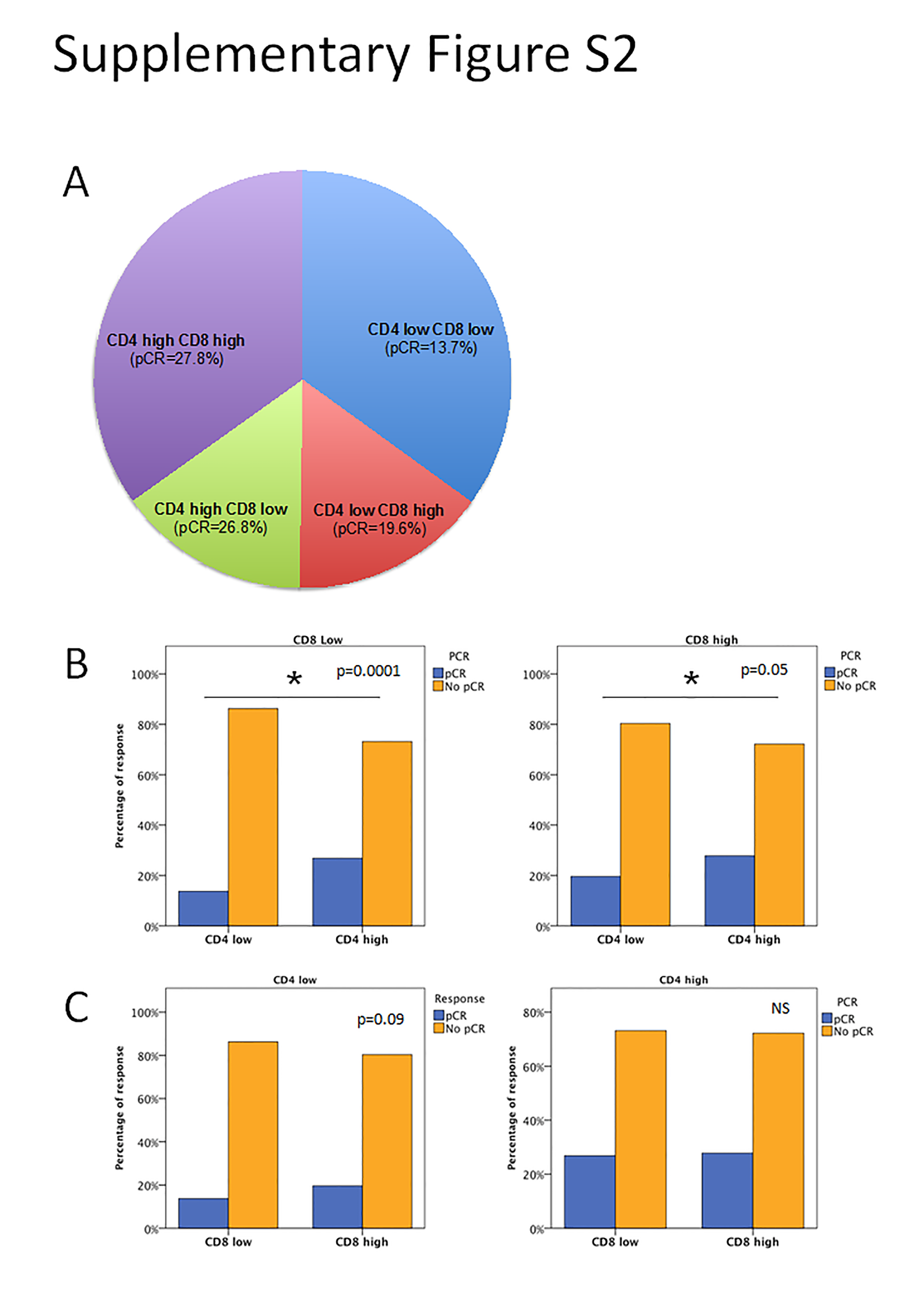

Supplement: Supplementary file 3 — Additional file 3: Figure S2: Relative contribution of CD4 and CD8 expression to pCR in public genomic datasets. A) distribution of cases among the whole series (n = 1001 patients; genomic datasets: GSE 16446, GSE 20194, GSE 20271, GSE 22093, GSE 23988, GSE 41998) according to high or low expression of CD4 and CD8 (cut-point: median value); pCR rates are shown for each subgroup. B) CD4 expression was associated with pCR both in the CD8 low group (P = 0.0001) and in the high CD8 group (P = 0.05). C) significant association of pCR with CD8 was found neither in the low CD4 group (P = 0.09) nor in the high CD4 expression group. Statistical analysis: χ2 test. *, P ≤0.05. NS, non significant. (TIFF 1 MB) [file 13058_2014_488_MOESM3_ESM.tiff]

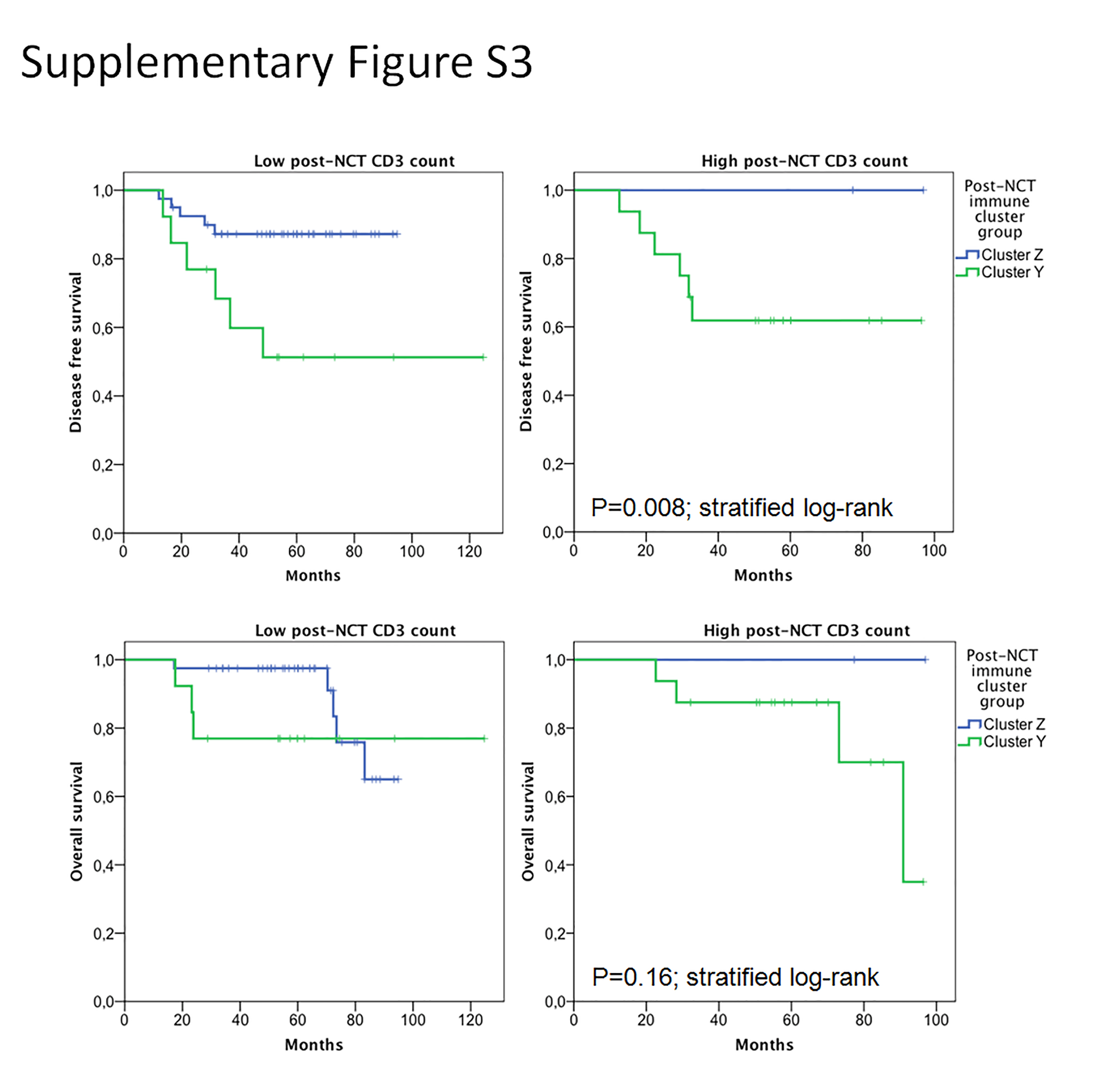

Supplement: Supplementary file 4 — Additional file 4: Figure S3: Disease free and overall survival analysis of post-NCT clusters (Y and Z) stratified by lymphocytic (CD3) infiltration. Kaplan-Meier curves showing the prognostic impact of post-NCT tumor-infiltrating immune cell profiles in tumors with low and high lymphocytic infiltration (defined as CD3 under or over P75). The difference was significant for DFS (P = 0.008; log-rank stratified by CD3 infiltration), while only a trend was found for OS (P = 0.16; log-rank). Stratified Wilcoxon test was significant both for DFS (P = 0.03) and OS (P = 0.03). (TIFF 1004 KB) [file 13058_2014_488_MOESM4_ESM.tiff]

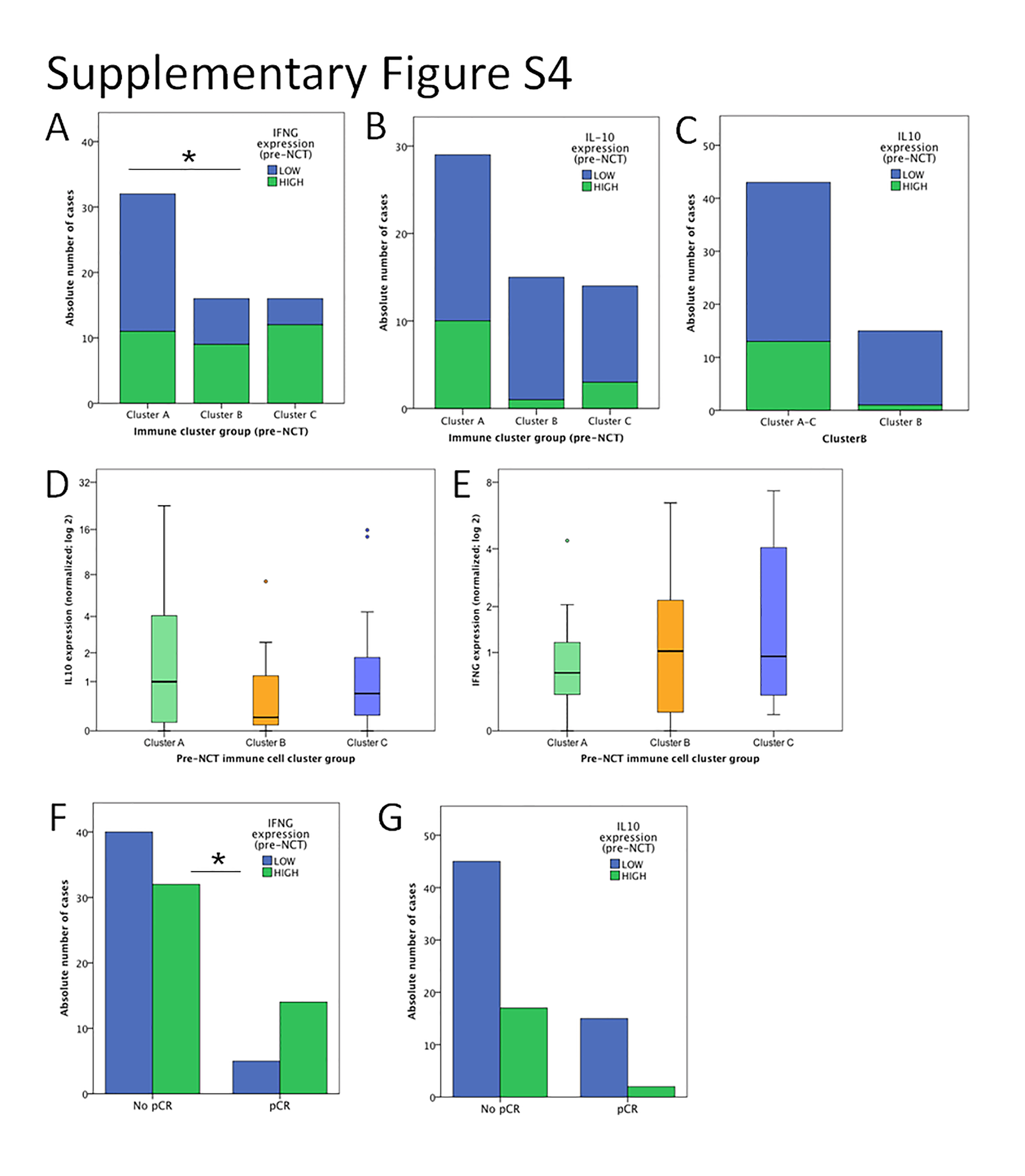

Supplement: Supplementary file 5 — Additional file 5: Figure S4: Pre-chemotherapy IFNG and IL10 expression. A) pattern of IFNG level of expression (over or below the median value) according to pre-NCT clusters (P = 0.025), B-C) pattern of IL10 expression (P75) according to pre-NCT clusters (P = 0.116; A-C versus B, P = 0.087). D) box-plot showing the level of IL10 expression among pre-treatment cluster groups (A-C); expression levels normalized to lower group (NS). E) level of IFNG expression according to pre-NCT cluster group (NS). F) association of IFNG level of expression (over or below median) with pCR (P = 0.023). G) association of IL10 expression (over or below P75) with pCR (NS). Statistical analysis: Mann–Whitney U-test or χ2 test . NS, non significant. *, P ≤0.05. (TIFF 1 MB) [file 13058_2014_488_MOESM5_ESM.tiff]

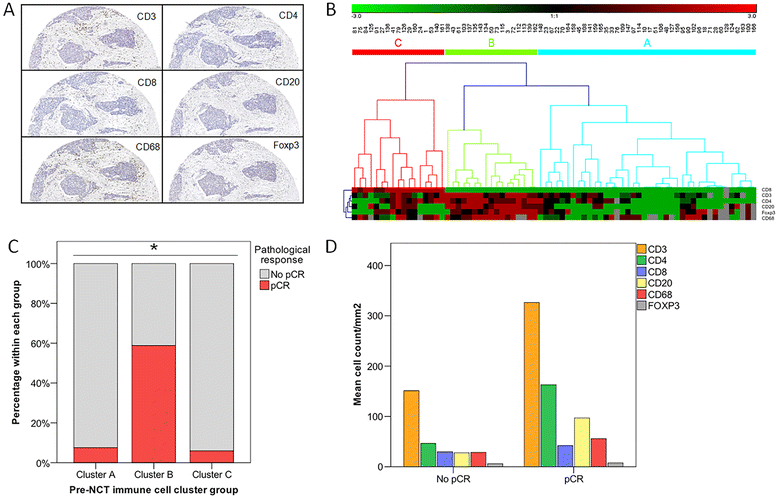

Supplement: Supplementary file 6 — Authors’ original file for figure 1 [file 13058_2014_488_MOESM6_ESM.gif]

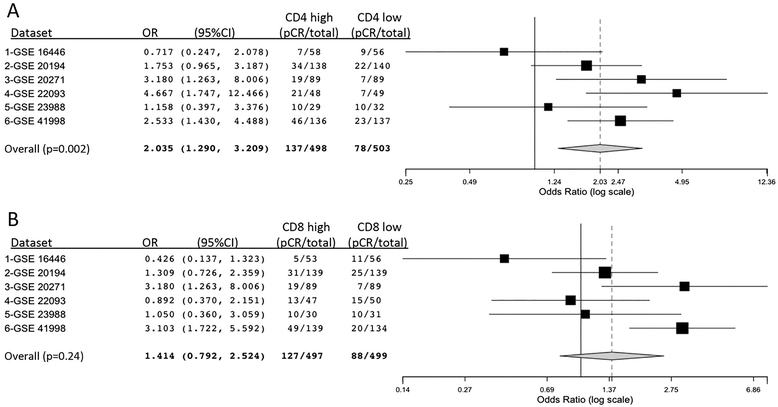

Supplement: Supplementary file 7 — Authors’ original file for figure 2 [file 13058_2014_488_MOESM7_ESM.gif]

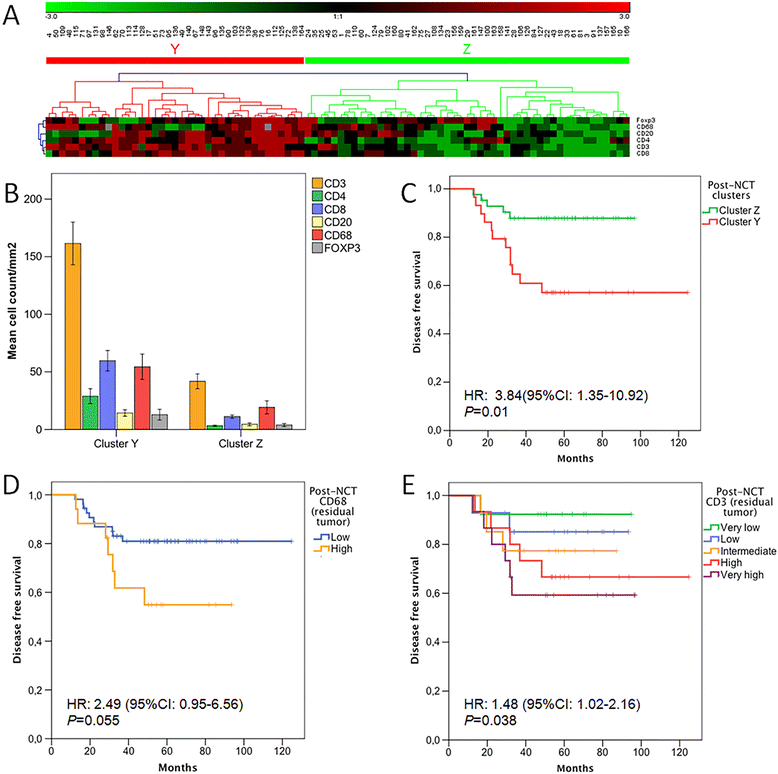

Supplement: Supplementary file 8 — Authors’ original file for figure 3 [file 13058_2014_488_MOESM8_ESM.gif]

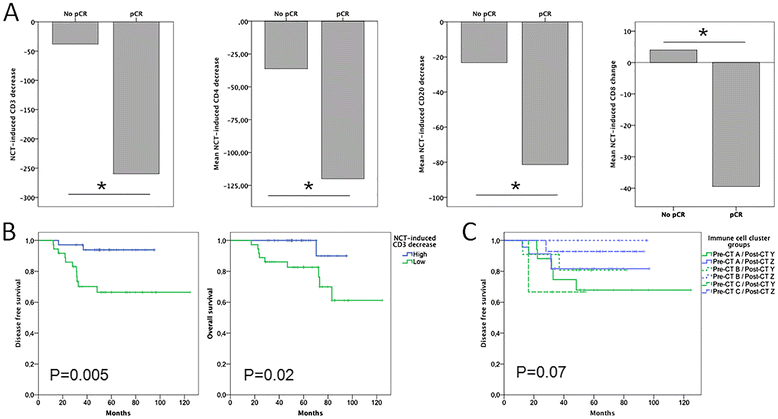

Supplement: Supplementary file 9 — Authors’ original file for figure 4 [file 13058_2014_488_MOESM9_ESM.gif]
