# Supplementary material for: Tumor-infiltrating immune cell profiles and their change after neoadjuvant chemotherapy predict response and prognosis of breast cancer
Source: Breast Cancer Res. 2014 Nov 29;16:488. doi: 10.1186/s13058-014-0488-5 (PMC4303200; doi:10.1186/s13058-014-0488-5)
Supplement: Supplementary file 2 — Additional file 2: Table S1: Distribution of TIL subpopulations according to breast tumor characteristics. Table S2. Association of post-chemotherapy immune cell infiltration with clinical and pathological characteristics. Table S3. Association of post-chemotherapy immune cell subpopulations with breast cancer disease-free (DFS) and overall survival (OS). Table S4. Prognostic effect (DFS) of post-treatment immune infiltrate in residual tumor according to tumor subtypes. Table S5. Association of HE-based classification of lymphocyte infiltration and immunohistochemistry-based assessment of CD3 and other immune cell subpopulations. Table S6. Impact of chemotherapy-induced changes (high versus low) on pCR (multivariate model including tumor phenotype). (PDF 176 KB) [file 13058_2014_488_MOESM2_ESM.pdf]

SUPPLEMENTARY TABLES

Supplementary Table S1. Distribution of TIL subpopulations according to breast tumor characteristics

	TIL/mm^2 pre-CT					
	**CD3/mm^2 pre-CT median±SD**	**CD4/mm^2 pre-CT median±SD**	**CD8/mm^2 pre-CT median±SD**	**FOXP3/mm^2 pre-CT median±SD**	**CD20/mm^2 pre-CT median±SD**	**CD68/mm^2 pre-CT median±SD**
Menopausal status						
Premenopausal	126.40 ± 213.74	65.16 ± 116.17	52.11 ± 136.19	3.82 ± 6.0	41.42 ± 86.61	36.83 ± 53.01
Postmenopausal	212.21 ± 410.67	69.23 ± 131.73	11.20 ± 52.86	9.94 ± 24.1	42.69 ± 80.84	31.29 ± 37.18
	p=0.47	p=0.32	p=0.15	p=0.82	p=0.98	p=0.83
Age						
<40 years	97.21 ± 93.95	17.38 ± 32.80	26.19 ± 40.89	3.55 ± 5.80	3.85 ± 6.85	20.49 ± 18.07
>40 years	100.99 ±121.57	75.26 ±131.15	30.90 ±108.77	7.49 ± 19.15	48.35 ± 88.07	35.95 ± 47.64
	p=0.80	p=0.09	p=0.04	p=0.56	p=0.13	p=0.62
Tumor type						
Ductal invasive	77.64 ± 344.64	69.47 ± 126.55	32.48 ± 105.36	7.47 ± 18.60	42.55 ± 83.83	33.81 ± 46.48
Lobular invasive	40.11 ± 52.48	1.05 ± 1.41	0.00 ± 0.00	0.79 ± 1.13	0.00 ± 0.00	28.05 ± 13.21
	p=0.45	p=0.16	p=0.45	p=0.32	p=0.07	p=0.65
Clinical staging						
IIA	69.80 ± 655.99	82.34 ± 141.24	39.69 ± 94.39	6.52 ± 20.37	14.01 ± 35.85	14.53 ± 18.19
IIB	167.04 ± 276.15	81.28 ± 146.92	20.65 ± 73.42	6.79 ± 8.42	58.78 ± 99.09	38.08 ± 46.98
IIIA	59.27 ± 80.58	56.23 ± 120.51	49.08 ± 149.87	7.34 ± 26.56	25.88 ± 73.23	41.56 ± 58.40
IIIB	61.52 ± 87.49	18.30 ± 34.77	27.95 ± 55.90	6.38 ± 8.12	4.20 ± 7.43	45.75 ± 45.91
IIIC	237.29 ± 237.73	65.67 ± 96.43	2.26 ± 7.31	7.19 ± 8.91	83.91 ±101.89	30.47 ± 35.51
	p=0.09	p=0.87	p=0.73	p=1.00	p=0.12	p=0.56
cT1-cT2	248.04 ± 467.68	65.81 ± 113.96	48.50 ±136.14	4.97 ± 13.29	52.18 ± 97.67	23.27 ± 37.92
cT3 -cT4	120.05 ± 186.55	68.39 ± 131.69	18.23 ± 70.50	8.45 ± 20.85	35.11 ± 71.69	41.83 ± 48.78
	p=0.24	p=0.44	p=0.77	p=0.42	p=0.48	p=0.02
cN0-cN1	186.92 ± 386.58	81.70 ± 141.51	31.03 ± 92.09	5.22 + 11.83	29.88 ± 70.01	31.98 ± 47.31
cN2-cN3	143.74 ± 200.88	39.74 ± 75.00	28.85 ± 119.75	10.39 +26.20	64.53 ±100.58	37.62 ± 41.40
	p=0.38	p=0.29	p=0.49	p=0.38	p=0.72	p=0.48
Tumor grade						
G I - II	176.03 ± 429.62	46.77 ± 98.57	21.38 ± 62.66	4.73 ± 13.01	13.37 ± 34.76	23.42 ± 26.50
G III	194.93 ± 254.43	96.22 ± 145.74	26.11± 91.36	10.14 ± 22.65	66.78 ± 99.00	46.47 ± 58.77
	p=0.72	p=0.45	p=0.40	p=0.94	p=0.07	p=0.22
Linfovascular invasion						
No	191.12 ± 361.43	70.51 ± 131.89	34.38 ± 111.34	7.59 ± 19.72	44.27 ± 84.91	36.56 ± 48.70
Yes	88.47 ± 151.15	51.16 ± 72.42	11.21 ± 31.08	4.06 ± 3.65	32.32 ± 76.57	23.15 ± 24.10
	p=0.23	p=0.90	p=0.66	p=0.29	p=0.56	p=0.64
Hormone-sensitivity						
No	205.93 ± 276.81	83.58 ± 144.89	40.14 ± 134.30	12.08 ± 26.44	72.59 ± 112.74	51.17 ± 50.02
Yes	158.54 ± 365.28	60.12 ± 113.03	25.64 ± 82.28	4.45 ± 11.17	26.07 ± 56.66	25.67 ± 41.00
	p=0.60	p=0.58	p=0.63	p=0.11	p=0.40	p=0.01
HER2 overexpression						
Negative	169.34 ± 359.42	51.19 ± 101.74	35.18 ± 112.50	6.22 ± 12.27	35.53 ± 78.08	27.10 ± 40.97
Positive	188.41 ± 277.85	109.73 ± 163.09	18.97 ± 71.98	9.07 ± 27.70	60.56 ± 95.53	50.68 ± 51.98
	p=0.65	p=0.25	p=0.58	p=0.83	p=0.14	p=0.06
IHQ subtype						
ER+ and/or PR+ and HER2-	180.37 ± 408.12	57.53 ± 112.98	31.09 ± 91.94	5.01 ± 12.55	26.86 ± 57.74	24.01 ± 43.15
ER+ and/or PR+ and HER2+	179.95 ± 103.04	68.58 ± 118.26	5.47 ± 14.82	2.58 ± 3.62	23.31 ± 55.56	31.39 ± 34.15
ER- and PR- and HER2+	296.86 ± 355.28	155.00 ± 198.05	32.46 ± 101.56	15.57 ± 38.90	97.81 ± 114.44	69.97 ± 61.15
ER- and PR- and HER2-	140.98 ± 193.08	35.97 ± 68.58	45.25 ± 155.63	9.39 ± 11.33	55.77 ± 112.30	35.79 ± 34.40
	p=0.52	p=0.10	p=0.83	p=0.33	p=0.08	p=0.06

Supplementary Table S2. Association of post-chemotherapy immune cell infiltration with clinical and pathological characteristics.

	TIL/mm² post-CT					
	CD3/mm² post-CT median±SD	CD4/mm² post-CT median±SD	CD8/mm² post-CT median±SD	FOXP3/mm² post-CT median±SD	CD20/mm² post-CT median±SD	CD68/mm² post-CT median±SD
**Menopausal status**						
**Premenopausal**	108.17 ± 105.43	16.82 ± 28.89	36.75 ± 50.76	25.05 ± 117.99	17.64 ± 74.36	47.06 ± 87.70
**Postmenopausal**	93.04 ± 128.61	26.92 ± 93.50	37.29 ± 79.78	7.58 ± 20.52	10.54 ± 13.99	31.29 ± 48.77
	p=0.07	p=0.16	p=0.42	p=0.96	p=0.07	p=0.94
**Tumor type**						
**Ductal invasive**	101.61 ± 120.54	22.79 ± 22.79	36.93 ± 68.28	16.82 ± 87.98	14.43 ± 55.41	39.09 ± 72.30
**Lobular invasive**	79.66 ± 45.35	6.59 ± 2.64	25.84 ± 15.16	12.76 ± 25.52	9.04 ± 16.03	39.75 ± 49.17
	p=0.88	p=0.64	p=0.67	p=0.21	p=0.55	p=0.58
**Clinical staging**						
**IIA**	88.29 ± 127.92	16.55 ± 36.67	23.88 ± 42.00	8.65 ± 24.25	5.77± 8.44	40.13 ± 78.92
**IIB**	87.87 ± 82.18	15.79 ± 30.44	34.20 ± 36.13	12.23 ± 28.65	11.53 ± 15.53	46.04 ± 89.69
**IIIA**	116.98 ± 124.14	13.40 ± 30.30	33.38 ± 57.95	5.94 ± 10.57	6.79 ± 9.95	45.70 ± 64.62
**IIIB**	91.81 ± 55.66	4.09 ± 4.08	21.86 ± 19.78	0.69 ± 0.98	9.84 ± 15.63	13.40 ± 14.99
**IIIC**	109.66 ± 166.46	58.69 ±153.67	65.99 ± 127.56	54.6 ± 202.90	41.07± 127.68	18.66 ± 25.34
	p=0.89	p=0.27	p=0.44	p= 0.45	p=0.32	p=0.70
**cT1-cT2**	109.67 ± 142.75	30.91 ± 100.78	42.55 ± 86.16	27.26 ± 128.17	22.38 ± 80.16	32.77 ± 60.34
**cT3 -cT4**	93.41 ± 94.75	15.20 ± 28.87	32.72 ± 46.66	8.35 ± 20.90	7.93 ± 14.57	43.75 ± 77.86
	p=0.76	p=0.84	p=0.77	p=0.68	p=0.23	p=0.27
**cN0-cN1**	97.52 ± 110.56	15.75 ± 33.05	33.12 ± 48.43	9.72 ± 23.88	9.72 ± 13.35	41.44 ± 78.10
**cN2-cN3**	106.68 ± 132.89	35.34 ±113.88	45.55 ± 95.68	31.07 ± 148.39	23.71 ± 93.97	33.83 ± 51.79
	p=0.73	p=0.87	p=0.74	p=0.45	p=0.13	p=0.84
**Tumor grade**						
**G I - II**	75.63 ± 94.83	9.46 ± 24.46	22.37 ± 33.50	5.95 ± 17.29	4.93 ± 6.63	24.63 ± 47.85
**G III**	117.40 ± 134.87	32.46 ± 91.95	48.69 ± 85.32	26.04 ± 115.85	21.60 ± 72.93	45.32 ± 80.17
	p=0.66	p=0.05	p=0.01	p=0.25	p=0.03	p=0.13
**Linfovascular invasion**						
**No**	104.14 ± 124.86	24.22 ± 75.29	39.19 ± 71.98	19.08 ± 93.17	16.11 + 58.58	42.01 ± 75.54
**Yes**	80.86 ± 65.18	10.17 ± 10.17	25.45 ± 20.13	2.94 ± 3.22	3.91 + 5.67	23.82 ± 34.96
	p=0.93	p=0.46	p=0.89	p=0.74	p=0.14	p=0.40
**Hormone-sensitivity**						
**No**	108.19 ± 125.85	37.18 + 108.88	46.62 ± 91.95	35.56 ± 142.02	24.67 ± 89.59	57.49 ± 94.89
**Yes**	95.26 ± 114.31	13.77 + 31.69	31.78 ± 48.69	6.02 ± 16.20	8.59 ± 10.89	26.81 ± 49.13
	p=0.35	p=0.39	p=0.39	p=0.76	p=0.32	p=0.11
**HER2 overexpression**						
**Negative**	96.67 ± 115.05	21.57 + 77.42	35.42 ± 68.81	18.48 ± 98.71	15.27 ± 62.09	38.83 ± 74.70
**Positive**	109.30 ± 128.61	23.69 + 42.64	41.77 ± 62.66	10.84 ± 24.30	11.52 ± 16.20	33.67 ± 53.58
	p=0.56	p=0.63	p=0.46	p=0.37	p=0.56	p=0.47
**IHC subtype**						
**ER+ and/or PR+ and HER2-**	83.29 ± 94.08	8.35 ± 20.93	26.34 ± 34.13	4.69 ± 13.95	6.89 ± 8.41	23.86± 43.36
**ER+and/or PR+ and HER2+**	144.22 ± 172.16	35.93 ± 54.07	54.52 ± 85.63	11.46 ± 23.38	15.54 ± 16.60	38.90 ± 69.32
**ER- and PR- and HER2+**	74.38 ± 49.13	11.45 ± 23.76	29.02 ± 23.58	10.21 ± 26.31	7.50 ± 15.50	28.44 ± 34.11
**ER- and PR- and HER2-**	126.78 ± 150.71	51.33 ± 133.52	56.29 ± 113.05	49.50 ±175.80	34.11 ± 110.83	74.31 ± 114.23
	p=0.26	p=0.11	p=0.29	p=0.27	p=0.30	p=0.06

Supplementary Table S3. Association of post-chemotherapy immune cell subpopulations with breast cancer disease-free (DFS) and overall survival (OS).

TIL post-CT > mean	OS (log-rank)	DFS (log-rank)
**CD3**	p=0.95	p=0.08
**CD4**	p=0.75	p=0.69
**CD8**	p=0.98	p=0.51
**FOXP3**	p=0.14	p=0.58
**CD20**	p=0.08	p=0.82
**CD68**	p=0.71	p=0.03
**CD4/CD8**	p=0.82	p=0.79
**FOXP3/CD3**	p=0.15	p=0.06
**FOXP3/CD8**	p=0.55	p=0.82
**CD68/CD8**	p=0.23	p=0.57

Supplementary Table S4. Prognostic effect (DFS) of post-treatment immune infiltrate in residual tumor according to tumor subtypes.

	HS*	HER2	Triple negative
**High vs Low**			
CD3	p=0.31	p=0.22	p=0.88
CD4	p=0.64	--	p=0.37
CD8	p=0.04	p=0.22	p=0.14
FOXP3	p=0.19	--	p=0.20
CD20	p=0.07	p=0.61	p=0.30
CD68	p=0.06	p=0.04	p=0.54
CD4/CD8	p=0.67	p=0.61	p=0.48
FOXP3/CD3	p=0.34	--	p=0.20
FOXP3/CD8	p=0.90	p=0.41	p=0.55
CD68/CD8	p=0.15	p=0.61	p=0.88

* Hormone-sensitivity

Supplementary Table S5. Association of HE-based classification of lymphocyte infiltration and immunohistochemistry-based assessment of CD3 and other immune cell subpopulations.

		Pre-NCT (Mean; SD) n=47		Post-NCT (Mean, SD) n=79	
		**CD3 count**	**Total count (CD3+CD20 +CD68)**	**CD3 count**	**Total count (CD3+CD20+ CD68)**
**HE groups**	No infiltration	79; 171	129; 180	38; 35	57; 52
	Partial infiltration	155; 192	228; 257	103; 76	150; 114
	Lymphocyte-predominant breast cancer	486, 201	721; 331	231; 217	298; 280
	P (Kruskal-Wallis)	0,00003	0,0003	0,0002	0,001

Supplementary Table S6. Impact of chemotherapy-induced changes (high vs low) on pCR (multivariate model including tumor phenotype).

**TIL change**	***P***	**0R**	**95%CI**
CD3	0.01	17.84	3.02 - 105.3
CD4	0.001	15.02	2.89 - 77.92
CD8	0.65	0.72	0.17 - 3.00
FOXP3	0.09	3.79	0.82 – 17.55
CD20	0.002	11.87	2.47 - 57.01
CD68	0.17	2.94	0.62 – 14.02
